# Selection of Immunobiotic *Ligilactobacillus salivarius* Strains from the Intestinal Tract of Wakame-Fed Pigs: Functional and Genomic Studies

**DOI:** 10.3390/microorganisms8111659

**Published:** 2020-10-26

**Authors:** Binghui Zhou, Leonardo Albarracin, Yuhki Indo, Lorena Arce, Yuki Masumizu, Mikado Tomokiyo, Md. Aminul Islam, Valeria Garcia-Castillo, Wakako Ikeda-Ohtsubo, Tomonori Nochi, Hidetoshi Morita, Hideki Takahashi, Shoichiro Kurata, Julio Villena, Haruki Kitazawa

**Affiliations:** 1 Food and Feed Immunology Group, Graduate School of Agricultural Science, Tohoku University, Sendai 980-8572, Japan; zhou.binghui.s5@dc.tohoku.ac.jp (B.Z.); lalbarracin@herrera.unt.edu.ar (L.A.); ex.windows84@gmail.com (Y.I.); lore.arce.oaq@gmail.com (L.A.); m.a.s.u.y.u.k.i02@gmail.com (Y.M.); mikado.tomokiyo.t4@dc.tohoku.ac.jp (M.T.); aminul.vmed@bau.edu.bd (M.A.I.); valeriagarcia@udec.cl (V.G.-C.); wakako.ohtsubo.a7@tohoku.ac.jp (W.I.-O.); 2 Livestock Immunology Unit, International Education and Research Center for Food Agricultural Immunology (CFAI), Graduate School of Agricultural Science, Tohoku University, Sendai 980-8572, Japan; tomonori.nochi.a5@tohoku.ac.jp; 3 Laboratory of Immunobiotechnology, Reference Centre for Lactobacilli (CERELA-CONICET), Tucuman 4000, Argentina; 4 Scientific Computing Laboratory, Computer Science Department, Faculty of Exact Sciences and Technology, National University of Tucuman, Tucuman 4000, Argentina; 5 Infection Biology Laboratory, INSIBIO-CONICET, Faculty of Medicine, University of Tucuman, Tucuman 4000, Argentina; 6 Department of Medicine, Faculty of Veterinary Science, Bangladesh Agricultural University, Mymensingh 2202, Bangladesh; 7 Laboratory of Functional Morphology, Graduate School of Agricultural Science, Tohoku University, Sendai 980-8572, Japan; 8 Graduate School of Environmental and Life Science, Okayama University, Okayama 700-8530, Japan; hidetoshi-morita@okayama-u.ac.jp; 9 Laboratory of Plant Pathology, Graduate School of Agricultural Science, Tohoku University, Sendai 980-8572, Japan; hideki.takahashi.d5@tohoku.ac.jp; 10 Plant Immunology Unit, International Education and Research Center for Food and Agricultural Immunology (CFAI), Graduate School of Agricultural Science, Tohoku University, Sendai 980-8572, Japan; 11 Laboratory of Molecular Genetics, Graduate School of Pharmaceutical Sciences, Tohoku University, Sendai 980-8572, Japan; shoichiro.kurata.d5@tohoku.ac.jp

**Keywords:** porcine intestinal epithelial cells, wakame, pigs, *Ligilactobacillus salivarius*, adhesion, immunomodulation, genomics

## Abstract

In this article, *Ligilactobacillus salivarius* FFIG strains, isolated from the intestinal tract of wakame-fed pigs, are characterized according to their potential probiotic properties. Strains were evaluated by studying their interaction with porcine intestinal epithelial (PIE) cells in terms of their ability to regulate toll-like receptor (TLR)-3- or TLR4-mediated innate immune responses, as well as by assessing their adhesion capabilities to porcine epithelial cells and mucins. These functional studies were complemented with comparative genomic evaluations using the complete genome sequences of porcine *L. salivarius* strains selected from subgroups that demonstrated different “immune” and “adhesion” phenotypes. We found that their immunomodulatory and adhesion capabilities are a strain-dependent characteristic. Our analysis indicated that the differential immunomodulatory and adhesive activities of FFIG strains would be dependent on the combination of several surface structures acting simultaneously, which include peptidoglycan, exopolysaccharides, lipoteichoic acid, and adhesins. Of note, our results indicate that there is no correlation between the immunomodulatory capacity of the strains with their adhesion ability to mucins and epithelial cells. Therefore, in the selection of strains destined to colonize the intestinal mucosa and modulate the immunity of the host, both properties must be adequately evaluated. Interestingly, we showed that *L. salivarius* FFIG58 functionally modulated the innate immune responses triggered by TLR3 and TLR4 activation in PIE cells and efficiently adhered to these cells. Moreover, the FFIG58 strain was capable of reducing rotavirus replication in PIE cells. Therefore, *L. salivarius* FFIG58 is a good candidate for further in vivo studying the protective effect of lactobacilli against intestinal infections in the porcine host. We also reported and analyzed, for the first time, the complete genome of several *L. salivarius* strains that were isolated from the intestine of pigs after the selective pressure of feeding the animals with wakame. Further genomic analysis could be of value to reveal the metabolic characteristics and potential of the FFIG strains in general and of the FFIG58 strain, in particular, relating to wakame by-products assimilation.

## 1. Introduction

The gastrointestinal tract harbors a dense and diverse microbial community capable of collaborating in key physiological processes of the host, such as supporting nutrient absorption, strengthening the intestinal barrier functions, and promoting mucosal immune system development [1,2,3]. The advancement of high-throughput sequencing technologies has allowed the culture-independent analysis of the composition and function of gut microbiota not only in humans, but also in animals of economic importance like pigs. Interestingly, the study of the porcine microbiota in different sections of the gastrointestinal tract described a low α-diversity and a high β-diversity in the small intestine of pigs when compared with the large intestine [2]. The higher microbial variability observed in the small intestine of adult pigs was attributed to the lower number of microorganisms present in this region, highlighting that this bacterial community is less stable and can be significantly affected by the influx of new bacteria from the feed. Thus, considering that the small intestine contains the largest population of immune cells in the organism, the regulation of the immune system through the modulation of the microbiota through the feed is an interesting alternative to improve the immune health status of the porcine host. In fact, the strengthening of a healthy immune system, which allows the efficient resistance against bacterial and viral infections minimizing unnecessary inflammation in the intestinal mucosa, has been associated with improved feed efficiency, feed conversion, feed intake, and body weight gain in pigs [4,5,6].

The use of immunomodulatory probiotics (immunobiotics) has been proposed as an alternative to improve growth performance and productivity in the swine industry, through the beneficial modulation of the immune system [7]. Thus, in the last decade, microbial strains isolated from the porcine gastrointestinal tract that can be used as probiotics/immunobiotics have been intensively sought [8,9]. Moreover, host-adapted probiotics, which are associated with a more efficient mucosal colonization, are considered more adequate to impart beneficial effects to the host. A study evaluating the microbial richness and diversity of the porcine intestinal tract at various growth stages demonstrated that *Bacteroides*, *Lactobacillus*, and *Prevotella* were the dominant genera in the gut of 10- to 63-days old commercial pigs, representing more than 40% of total 16S rRNA gene sequences [1]. In addition, the analysis of the gut microbiota in 120-days old pigs showed that the most abundant genus in the duodenum was *Lactobacillus* [2]. Then, strains of the genus *Lactobacillus* are considered promising probiotic candidates for pigs. In this regard, *Ligilactobacillus salivarius* (Basonym: *Lactobacillus salivarius*) [10], has been characterized as a normal member of the porcine intestinal microbiota [11] and has been proposed as a beneficial species with probiotic potential [12,13].

In a recent study, we demonstrated that feeding pigs with wakame (*Undaria pinnatifida*), a popular and economically important edible algae in Asian countries [14], was able to modify the gastrointestinal microbiota inducing a significant increase in the abundance of *L. salivarius* [15]. Moreover, considering the reports that indicate that wakame feeding was associated with a beneficial modulation of the pigs’ immune system [16]; we hypothesized that the increase in lactobacilli would be associated with the immunomodulatory effect of wakame. Then, by using wakame-based mediums originally developed by our group [15], we constructed a library of *L. salivarius* strains isolated from wakame-fed pigs. We also investigated the capacities of lactobacilli strains to modulate interferon (IFN)-β expression in response to toll-like receptor (TLR)-3 activation in porcine intestinal epithelial (PIE) cells. Our results demonstrated a strain-dependent ability to improve IFN-β in PIE cells after TLR3 activation [15].

In this work, we aimed to further advance in the characterization of the potentially beneficial properties of *L. salivarius* strains isolated from the intestine of wakame-fed pigs by evaluating their interaction with PIE cells in terms of their ability to regulate TLR3- or TLR4-mediated innate immune responses, as well as their adhesion capabilities. The functional studies were complemented with a comparative genomic evaluation using the complete genome sequences of porcine *L. salivarius* strains selected from subgroups that demonstrated different “immune” and “adhesion” phenotypes.

## 2. Materials and Methods

### 2.1. L. salivarius Strains

*L. salivarius* strains were isolated from the mucus membrane of the small intestine (jejunum, jejunum Peyer’s patches, ileum, and ileum Peyer’s patches) of wakame-fed pigs by using originally developed wakame-based mediums as described previously [15]. The lactobacilli strains and their isolation origin evaluated in this work are listed in Table S1. The *L. salivarius* strains isolated from the intestinal tract of wakame-fed pigs were designated as FFIG.

For the experiments of this work, lactobacilli strains were grown in Man–Rogosa–Sharpe (MRS) broth at 37 °C. For the in vitro immunomodulatory assays, overnight cultures were harvested by centrifugation, washed three times with sterile phosphate-buffered saline (PBS), counted in a Petroff–Hausser counting chamber, and resuspended in DMEM until use.

### 2.2. PIE Cells 

The PIE cell line was originally established at Tohoku University from the intestinal epithelia of an unsuckled neonatal pig, as described previously [17,18]. DMEM medium supplemented with 10% fetal calf serum (FCS), penicillin (100 mg/mL), and streptomycin (100 U/mL) was used for the maintenance of PIE cells. The cells (3.0 × 10^4^ per well) were grown in 12 well type I collagen-coated plates at 37 °C in a humidified atmosphere of 5% CO_2_. After three days of culturing period, 1 mL of DMEM containing the different *L. salivarius* strains isolated from the intestine of wakame-fed pigs (5 × 10^7^ cells/mL) were added to PIE cells monolayers. Cells were further incubated for 48 h at 37 °C, 5% CO_2_. PIE cells were washed with fresh medium to eliminate lactobacilli and subsequently stimulated with 10 ug/mL of poly(I:C) (Sigma Aldrich, St. Louis, MI, USA) or enterotoxigenic *Escherichia coli* (ETEC) for 12 h, to induce the activation of TLR3 and TLR4, respectively. The expressions of IFN-β and Mx1 were evaluated after TLR3 activation, while the expression of IL-8 and MCP-1 were studied after TLR4 stimulation.

### 2.3. RT-qPCR

The expression of immune factors in PIE cells was studied as described previously [17,18,19]. Briefly, total RNA was extracted with TRIzol reagent (Invitrogen, Carlsbad, CA, USA), and its purity and quantity were analyzed by a NanoDrop spectrophotometer ND-1000 UV-Vis (NanoDrop Technologies, Wilimington, DE, USA). The RNA (500 ng) was used to synthesize cDNA by Thermal cycler (BIO-RAD, Hercules, CA, USA) with the Quantitect reverse transcription (RT) kit (Qiagen, Tokyo, Japan) following the manufacturer instructions. The qPCR was performed in a 7300 real-time PCR system (Applied Biosystems, Warrington, UK) with platinum SYBR green (qPCR supermix uracil-DNA glycosylase with 6- carboxyl-X-rhodamine, Invitrogen). For the PCR reaction, 2.5 μL of cDNA were mixture with 7.5 μL of the master mix that included RT enzyme, SYBR green, forward, and reverse primers (1 pmol/μL). The reaction cycles were performed as follow: 50 °C for 5 min; 95 °C for 5 min; 40 cycles at 95 °C for 15 s, 60 °C for 30 s and finally 72 °C for 30 s. β-actin was used as a housekeeping gene because of its high stability across various porcine tissues [17,18,19]. The expression of the housekeeping gene was used to normalize cDNA levels for differences in total cDNA levels in the samples.

### 2.4. Purification of Porcine Intestinal Mucins

Porcine intestinal mucins were used to evaluate the adhesion of FFIG strains [20]. Crude mucus was scraped from porcine small intestinal tissue. Mucus was digested with 0.5 mg/mL of proteinase K (TaKaRa Biotechnology, Shiga, Japan) overnight. After centrifugation (8500× *g*, 4 °C, 10 min) and membrane filtration (DISMIC-25, 0.45 μm, Advantec, Tokyo, Japan), the supernatant was purified by gel filtration chromatography with a Toyopearl HW-65F column (90 × 2.6 cm; Tosoh, Tokyo, Japan) using distilled water as the mobile phase. Peptides were detected at 214 nm, and neutral sugar was measured at 490 nm using the phenol-sulfuric acid method. Fractions containing high concentrations of sugars and peptides were collected and concentrated before lyophilization. The purified soluble porcine mucins were used as ligands for the Biacore analysis.

### 2.5. Biacore Assay

Biacore experiments were performed using a Biacore 1000 (GE Healthcare Bio-Sciences K.K., Sheffield, UK) at 25 °C in ab HBS-EP buffer [21]. The immobilization of purified porcine mucins on a CM5 sensor chip (GE Healthcare Bio-Sciences K.K.) was induced by an amine coupling reaction following the manufacturer’s instructions. Mucins were dissolved at a concentration of 10 mg/mL in 10 mM sodium acetate buffer (pH 4.0) and immobilized using the reaction between N-hydroxysuccinimide (NHS)-esters and radicals of primary amino groups present in mucins molecules. The sensor chip was equilibrated in an HBS-EP buffer.

Adhesion using the Biacore 1000 is based on the principle of surface plasmon resonance. After washing and lyophilization, bacterial cells were suspended in an HBS-EP buffer (3 mg/mL). The bacterial suspension was injected at a flow rate of 3 μL/min for 5 min, the sensor chip was washed with HBS-EP buffer to remove unbound analyte; and regenerated eluting with 1 M guanidine hydrochloride (GHCl) solution at a flow rate of 3 μL/min for 2 min. The resonance units (RU) were measured for 200 s after the cessation of sample addition. A response of 1 RU represents 1 pg/mm^2^ protein adhering with an increased concentration of analyte bound to the sensor chip surface.

### 2.6. Adhesion to PIE Cells

The adhesion of lactobacilli to PIE cells was performed by the microplate method using fluorescent bacteria [22]. Cultured lactobacilli were washed with PBS three times (6000 rpm, 10 min). Pellet was resuspended in 1 mL PBS, and 1 mM of carboxyfluorescein diacetate (CFDA) was added for the fluorescent reaction at 37 ℃ for 1 h. Then, bacteria were washed with PBS three times (6000 rpm, 10 min) to remove CFDA on the microbial surface. Fluorescent bacteria were counted by hemocytometer.

PIE cells were seeded at 5000 cells/well in Type I collagen-coated 96 well cell culture plate (Nippi Incorporated, Tokyo) for 3 days. Cultured fluorescent lactobacilli were added to PIE cells at 100 MOI and co-cultured for 48 h. After incubation, non-adherent bacteria were washed out with PBS. After lysis with 0.1 N NaOH, fluorescence was evaluated by 2030 Multilabel Reader (Perkin Elmer, Fukuoka, Japan).

### 2.7. Scanning Electron Microscopy (SEM) Analysis

Lactobacilli were washed once and diluted 2 times with PBS. The suspension with bacteria was dropped on the polycarbonate membrane (ADVANTEC) and filtered with vacuum filtration (Miripore). The membrane with lactobacilli on the surface was immersed in 2% (*v*/*v*) glutaraldehyde solution. After 1 h, the membrane was immersed in 50, 60, 70, 80, 90, and 99% ethanol for 20 min at a time to remove water. The membrane was immersed in t-butyl alcohol, lyophilized, and treated with platinum palladium. SEM observation was performed in a HITACHI microscope.

### 2.8. Complete Genome Sequencing

The complete genome sequence of *L. salivarius* FFIG58 was reported recently [23]. Seven additional FFIG strains were selected for complete genome sequencing in this work. Single colonies of the selected *L. salivarius* *FFIG strains* cultured on MRS (Oxoid, Cambridge, UK) agar plates were inoculated separately into MRS broth and incubated at 37 °C for 12 h. The isolation of the genomic DNA was performed by using a lysozyme lysis buffer (75 mM NaCl, 20 mM EDTA, 20 mM Tris-HCl, pH 7.5, 10 mg/mL lysozyme), the chloroform-isoamyl alcohol separation, and isopropanol precipitation method as described previously [23,24]. The genomic DNA samples were sequenced with an Illumina HiSeq platform using the 2 × 300-bp paired-end read length-sequencing protocol. The paired-end sequencing libraries were prepared using the TruSeq DNA HT Sample Prep Kit (Illumina) according to the manufacturer’s protocol. The paired-end reads were filtered with PrinSeq-lite (v.0.20.4) and assembled with A5-miseq (v.201690825) with default parameters [23]. 

Sequencing results were analyzed using various software programs at their default settings, unless otherwise specified. Gene prediction and annotation were performed using NCBI Prokaryotic Genome Annotation Pipeline (PGAP) (v.4.12) [25] and Rapid Annotations using Subsystems Technology (RAST) [26]. 

### 2.9. Bioinformatic Analysis

The genome sequences of *L. salivarius* were downloaded from the GenBank database (https://www.ncbi.nlm.nih.gov/genome/1207). The average nucleotide identity (ANI) was calculated using the Enveomics collection web [27]. Phylogenetic trees were constructed to examine the relationships between the different microorganisms. The gene sequences were downloaded from the GenBank databases. The MUSCLE aligner [28] available in the MEGAX (v.10.0.4) [29] software, was employed to align the gene sequences of all microbes before the construction of the phylogenetic tree according to the Neighbor-Joining (NJ) distance algorithm [30,31] embedded in the MEGAX software as well. Heat-map figures were constructed using tools for plotting data [32] in R scripts. Pangenome analysis was conducted with Roary (v.3.6.0) [33] using the Prokka annotation (v.1.12) [34]. De Venn diagrams were generated with InteractiVenn [35].

### 2.10. Rotavirus Infection

A rotavirus strain isolated from pigs (OSU) was used in this study. Obtention of rotavirus for infection experiments was performed as described previously [36]. Briefly, rotavirus OSU was treated with 10 μg/mL trypsin (Sigma, Type I, St. Louis, MI, USA) at 37 °C for 30 min and inoculated onto confluent MA104 cells. After an hour of absorption, the inoculum was removed, and the cells were incubated with serum-free MEM containing 1 μg/mL trypsin at 37 °C. When the cytopathic effect reached more than 80%, the culture supernatant was harvested by three rounds of freezing and thawing process. The virus stock was stored at −80 °C for further experiments.

PIE cells were plated at 3.0 × 10^4^ cells/well in 12 well type I collagen-coated plates (SUMILON, Tokyo, Japan) and incubated at 37 °C, 5% CO_2_. After eight days of culturing, cells were pre-stimulated with *L. salivarius* FFIG58 isolated from the intestine of wakame-fed pigs (5 × 10^7^ cells/mL). Then, cells were washed three times with DMEM medium to eliminate the bacteria and subsequently inoculated with trypsin-activated rotavirus OSU at a multiplicity of infection (MOI) 1. At hour 16 post-inoculation, PIE cells were fixed after removing the inoculums, and the infected virus titer was analyzed by immunofluorescence staining.

The immunofluorescence staining for detecting cells infected with rotavirus was performed as described previously [36]. Briefly, rotavirus challenged-PIE cells were fixed with 80% acetone at 4 °C for 15 min. Then, cells were washed twice with PBS, and subsequently incubated with a guinea pig anti-rotavirus Wa strain polyclonal antibody (1:750 in PBS, 50 μL/well) for 30 min at 37 °C. After three washes with PBS, cells were incubated at 37 °C for 30 min with Fluorescein isothiocyanate (FITC) conjugated anti-guinea pig IgG (H+L) antibody (Rockland antibodies and assays, Limerick, PA, 1:400 in PBS, 50 μL/well). Infected cells were examined and photographed under an immunofluorescence microscope (Confocal laser microscope, MRC-1024, Bio-Rad, Richmond, CA) after three rounds of washing with PBS and mounted with 30% glycerol prepared in PBS. In addition, the expressions of IFN-β and Mx1 were determined by RT-qPCR, as described above, after 12 h of rotavirus infection.

### 2.11. Statistical Analysis

Statistical analyses were performed using the GLM and REG procedures available in the SAS computer program (SAS, 1994). Comparisons between mean values were carried out using one-way analysis of variance and Fisher’s least-significant-difference (LSD) test. For these analyses, *p* values of < 0.05 were considered significant.

## 3. Results and Discussion

### 3.1. Characterization of the Ability of L. salivarius Isolated from the Intestinal Tract of Wakame-Fed Pigs to Modulate Innate immune Responses in PIE Cells

**Figure 1:**
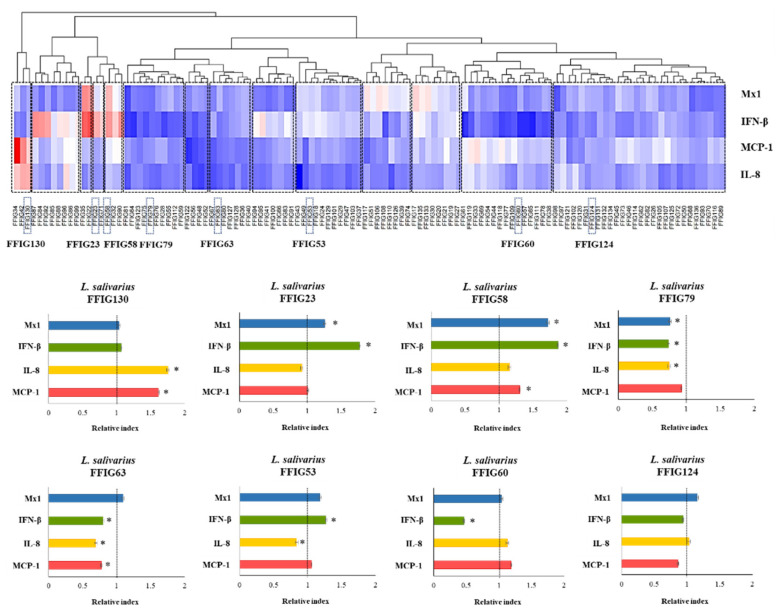
Modulation of toll-like receptors (TLRs) mediated innate immune responses in porcine intestinal epithelial (PIE) cells by *Ligilactobacillus salivarius* strains isolated from the intestinal mucosa of wakame-fed pigs. PIE cells were stimulated with the different *L. salivarius* strains and challenged with poly(I:C) or enterotoxigenic *Escherichia coli* (ETEC) to induce the activation of TLR3 and TLR4, respectively. The expressions of interferon (IFN)-β, and the antiviral factor Mx1 were analyzed by qPCR after 12 h of TLR3 activation. The expression of interleukin (IL)-8 and monocyte chemoattractant protein 1 (MCP-1) were analyzed by qPCR after 12 h of TLR4 activation. The results represent data from three independent experiments. Asterisks indicate significant differences when compared to the expression of immune factors in control PIE cells, which were set as one (* *p* < 0.05). Heat-map was constructed considering the fold changes relative to the control PIE cells not treated with lactobacilli and stimulated with poly(I:C) or ETEC.

One-hundred and sixteen *L. salivarius* strains isolated from the jejunum, ileum, or ileum Peyer’s patches of wakame-fed pigs (Table S1) were evaluated according to their ability to modulate the response of PIE cells to the challenges with TLR3 or TLR4 ligands (Figure 1). The expression levels of IFN-β and Mx1 were evaluated after TLR3 activation, while the activation of TLR4 was assessed by determining IL-8 and MCP-1. We observed that *L. salivarius* strains isolated from the intestine of wakame-fed pigs modulated the response of PIE cells in a strain-dependent manner. The comparative analysis of IFN-β, Mx1, IL-8, and MCP-1 expression changes clearly showed the different capacities of *L. salivarius* strains in modulating the innate immune responses in the epithelial cells of porcine origin (Figure 1). The different effects induced by the strains allowed them to be grouped into 14 groups, as shown in the clusters of the heat-map analysis. Strains like *L. salivarius* FFIG58 and FFIG23 showed a remarkable ability to improve the expression of IFN-β and Mx1 in PIE cells after TLR3 activation. Of note, these two strains clustered in different groups, since FFIG58 was also capable of improving MCP-1 expression in ETEC-challenged PIE cells, while the FFIG23 strain did not induce effects in IL-8, or MCP-1 expression after TLR4 activation. *L. salivarius* FFIG53 was also capable of increasing the expression of IFN-β, but no effect was observed for Mx1 (Figure 1). 

In contrast to FFIG58, FFIG23, and FFIG53, strains, such as FFIG60, FFIG63, and FFIG79, significantly reduced the expression of the type I IFN in poly(I:C)-challenged PIE cells. *L. salivarius* FFIG79 not only decreased IFN-β, but also reduced the expression of Mx1, an effect that was not observed for the FFIG60 or FFIG63 strains. The strains FFIG60, FFIG63, and FFIG79 also differed in their abilities to modulate IL-8 and MCP-1 in ETEC-challenged PIE cells. *L. salivarius* FFIG63 reduced the expression of both inflammatory factors; the FFIG79 diminished only IL-8 expression, while the FFIG60 did not modify the levels of IL-8 or MCP-1 (Figure 1). We also found strains with no ability to influence the response of PIE cells to TRL3 activation, but with the capacity to increase the expression of IL-8 or MCP-1 after ETEC challenge, such as *L. salivarius* FFIG130. In addition, strains unable to modulate neither the TLR3- nor the TLR4-mediated responses in PIE cells, such as *L. salivarius* FFIG124, were also detected (Figure 1).

Our previous studies evaluating the immunomodulatory potential of lactobacilli in PIE cells demonstrated that *Lacticaseibacillus casei* MEP221104 (Basonym: *Lactobacillus casei* MEP221104) improved the expression of IL-1α, IL-6, IL-8, and MCP-1 in ETEC-challenged PIE cells more efficiently than *L. casei* MEP221106. Similarly, *Lacticaseibacillus rhamnosus* MEP221111 (Basonym: *Lactobacillus rhamnosus* MEP221111) was more efficient in the induction of IL-6 and MCP-1 in PIE cells after the activation of TLR4 when compared with *L. rhamnosus* MEP221112 [37]. On the other hand, we demonstrated that both *Lactiplantibacillus plantarum* MPL16 (Basonym: *Lactobacillus plantarum* MPL16) and *L. plantarum* CRL1506 were capable of increasing the expression of IFN-β and the antiviral factors Mx2 and RNAseL in poly(I:C)-challenged PIE cells although the MPL16 was more efficient that the CRL1506 strain to induce this effect. Of note, when the two strains were compared in their ability to modulate the innate antiviral immune response in vivo in a mouse model, *L. plantarum* MPL16 was more efficient than the CRL1506 strain to increase the intestinal levels of IFN-β and IFN-γ, reduce TNF-α and IL-15 and to protect against the TLR3-mediated inflammatory damage [18,38]. In line with our previous investigations, the results obtained in this work indicate that the capacity of the porcine *L. salivarius* strains to differentially modulate the antiviral factors response activated by poly(I:C) stimulation or the production of inflammatory cytokines, induced by ETEC challenge in PIE cells, are strain-dependent characteristics. 

Even the FFIG strains were isolated from the same niche and under the same environmental pressure (wakame as the main nutritive substrate); they showed different capacities to modulate innate immune responses in PIE cells. This implies that detailed studies of their potentially beneficial properties are necessary to select the strains with the greatest capacity to positively influence the health of the porcine host. Furthermore, the comparative study of strains with different immunomodulatory capacities could provide key information to understand the mechanisms involved in their beneficial effects, as well as to select biomarkers that can be used in the future to quickly and efficiently select new probiotic strains. Then, taking into account the different capacity of the *L. salivarius* strains isolated from the intestine of wakame-fed pigs to modulate innate immunity in PIE cells, we selected eight strains (FFIG23, FFIG53, FFIG58, FFIG60, FFIG63, FFIG79, FFIG124, FFIG130) belonging to different clusters (Figure 1) for further functional and genomic studies aimed at characterizing their potential probiotic properties.

### 3.2. General Genomic Features of L. salivarius Isolated from the Intestinal Tract of Wakame-Fed Pigs

To deepen the characterization of the selected *L. salivarius* strains, their complete genomes were sequenced by Illumina HiSeq. The genome of *L. salivarius* FFIG58 was annotated and published recently [23], while the genomes of FFIG23, FFIG53, FFIG60, FFIG63, FFIG79, FFIG124, and FFIG130 were sequenced for this work (Table 1). The FFIG58 draft genome sequence has an average GC content of 32.9% and a total estimated size of 1,984,180 bp (Table 1). The other *L. salivarius* strains isolated from the intestinal tract of wakame-fed pigs showed general genomic features that were similar to those found for the FFIG58. The largest genome size was found in *L. salivarius* FFIG23 with 2,041,027 bp, while the smallest genome size was found in the FFIG79 strain with 1,718,597 bp. The average GC content in all the strains ranged between 32.8 and 33.4% (Table 1).

The clustering of pair-wise average nucleotide identity (ANI) was used as a method to confirm that the FFIG strains belong within the *L. salivarius* species, using a cut-off value of 95% as the species boundary [39]. The heat-map analysis of ANI values of FFIG strains compared with several *L. salivarius* strains clearly indicated that they belong to this species (Figure S1).

**Figure 2:**
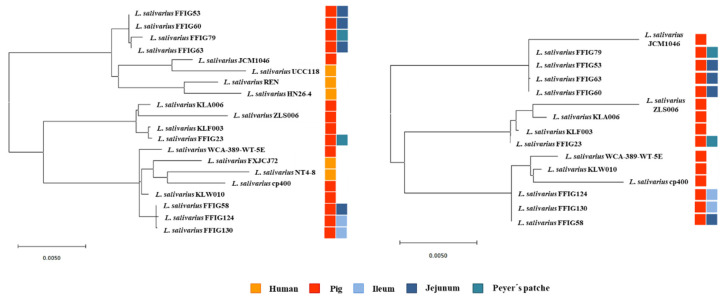
Molecular phylogenetic analysis by Maximum Likelihood method of *Ligilactobacillus salivarius* strains isolated from the intestinal mucosa of wakame-fed pigs. The phylogenetic trees were constructed based on the MLST analysis by using the genes *parB*, *rpsB*, *pheS*, *nrdB*, *groEL*, and *ftsQ* present the genomes of FFIG strains, as well as in *L. salivarius* strains with available public genomes.

To verify that the FFIG isolates were different strains, we constructed phylogenetic trees using the sequences of the 16s rRNA genes (Figure S2) and the Maximum Likelihood method (MLST) analysis with the sequences of the genes *parB*, *rpsB*, *pheS*, *nrdB*, *groEL*, and *ftsQ* (Figure 2) [11,40]. In both analyses, the FFIG isolates were compared with *L. salivarius* strains isolated from the intestinal tract of humans (UCC118, REN, HN26-4, NT4-8, and FXJCJ72) or pigs (JCM1046, ZSL006, cp400, KLA006, KLF003, KLW010, and WCA-389-WT-5E), and with available public genomes.

The analysis of the phylogenetic tree constructed with the sequences of the 16s rRNA genes revealed that the FFIGs strains clustered in different positions and together with most of the *L. salivarius* strains of the porcine origin (Figure S2). The most remarkable difference was found for *L. salivarius* FFIG79 that showed a significant distance when compared to the other strains of porcine origin, including the FFIG strains. The MLST analysis that included both human and porcine strains revealed that the FFIG strains clustered in three groups: One containing the FFIG53, FFIG60, FFIG63, and FFIG79 strains, another containing the FFIG58, FFIG124, and FFIG130 strains, while the FFIG23 clustered alone and close to the porcine strains KLF003, KLA006, and ZLS006. These groups were not modified when human strains were excluded from the MLST analysis (Figure 2).

The general genomic characteristics of the FFIGs strains (Table 1, Table S2) were not different from the reported for the genomes of *L. salivarius* strains available in public databases (Table S3). However, it was noted that the average genome sizes of the FFIG strains were smaller than those found for other strains of porcine origin. The genome sizes of *L. salivarius* JCM1046, ZSL006, cp400, KLA006, KLF003, and KLW010 ranged from 1,836,297 to 2,389,395 bp. The number of protein-coding genes ranged from 1803 in the JCM1046 strain to 2276 in the KLA006 strain (Table S3), while for the FFIG strains, the number of protein-coding genes ranged from 1726 (FFIG79) to 1932 (FFIG23) (Table 1).

### 3.3. Comparative Genomic Analysis of L. salivarius Isolated from the Intestinal Tract of Wakame-Fed Pigs Belonging to Different “Immune Phenotypes”

**Figure 3:**
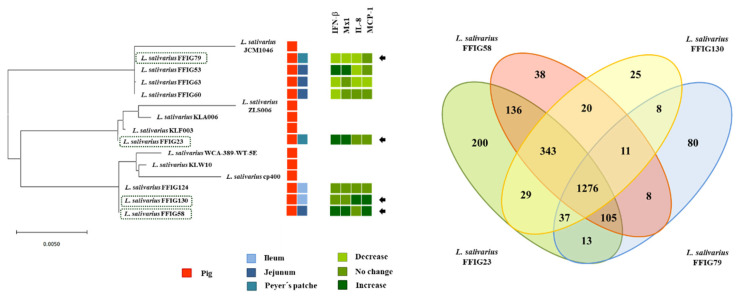
Genomic comparison of *Ligilactobacillus salivarius* strains isolated from the intestinal mucosa of wakame-fed pigs. Four “immune phenotypes” were defined according to the ability of *L. salivarius* FFIG strains of modulating TLRs mediated innate immune responses in porcine intestinal epithelial (PIE) cells. The phylogenetic tree constructed with the MLST analysis of the genes *parB*, *rpsB*, *pheS*, *nrdB*, *groEL*, and *ftsQ* is used to show the strains. *L. salivarius* FFIG23, FFIG58, FFIG79, and FFIG130 were compared. Venn diagram depicts the number of unique genes in each genome and the numbers of genes sheared by the strains.

We next carried out comparative genomic studies to characterize the *L. salivarius* FFIG strains with different immunomodulatory activities. Thus, we defined four principal “immune phenotypes”, according to the ability of the FFIGs strains to modulate the innate immune response in PIE cells after TLR3 or TLR4 activation (Figure 3). Strains with the ability to increase IFN-β and Mx1 and with no remarkable effect on IL-8 and MCP-1 expressions, such as FFIG23 and FFIG53, and strains with the capacity to reduce IFN-β and IL-8 and/or MCP-1, such as FFIG60, FFIG63, and FFIG79 were separated in two “immune phenotype” groups. In addition, the FFIG58 strain capable of increasing IFN-β, Mx1, and MCP-1, and the FFIG130 able to increase IL-8 and MCP-1, but with no effect on IFN-β and Mx1 were considered two “immune phenotype” groups. The FFIG124 with no effects on the expression of IFN-β, Mx1, IL-8, or MCP-1 in PIE cells was also included as a non-immunomodulatory strain.

The comparative study of four strains (FFIG58, FFIG23, FFIG130 and FFIG79) belonging to each of the “immune phenotype” groups revealed a coregenome of 1276 genes (Figure 3). The strains with the ability to increase IFN-β, *L. salivarius* FFIG58, and FFIG23 had 38 and 200 unique genes, respectively. In addition, the FFIG58 and FFIG23 strains sheared 136 genes that were not found in the FFIG130 or FFIG79 genomes.

Among the unique genes of *L. salivarius* FFIG58, we found a peptidoglycan O-acetyltransferase (*patA3*), a septation ring formation regulator (*ezrA*), a membrane protein YdfK (*ydfK*), and an N-acetyl-LL-diaminopimelate aminotransferase (*dapX*) (Table S4). The unique genes for FFIG23 strain included a peptidoglycan O-acetyltransferase (*patA1*), a glycosyltransferase EpsH (*epsH*), and an α-galactosylglucosyldiacylglycerol synthase (*cpoA*) (Table S4). Among the genes sheared by FFIG23 and FFIG58 we found the inner membrane protein YhaI (*yhaI*), the inner membrane transporter YicL (*yicL*), the glycosyltransferase EpsD (*epsE*), the penicillin-binding protein PbpX (*pbpX*), the membrane protein insertase MisCB (*misCB*) and the UDP-N-acetylenolpyruvoylglucosamine reductase (*murB*).

*L. salivarius* FFIG130, the strain with the ability to increase IL-8 and MCP-1, had 25 unique genes. In addition, FFIG130 sheared with the FFIG58 strain 20 genes that were not found in FFIG23 or FFIG79 strains (Figure 3). Among the unique genes of FFIG130 there was the peptidoglycan O-acetyltransferase (*patA5*), the cell division topological determinant MinJ (*minJ*), and an N-acetylmuramoyl-L-alanine amidase domain-containing protein different from the one found in the FFIG58 strain (Table S4). On the other hand, *L. salivarius* FFIG79 had 80 unique genes that included an inner membrane transport permease YbhR (*ybhR*). In addition, it was observed that FFIG79 sheared with FFIG23 a glycosyltransferase EpsJ (*epsJ*) that was not found in the other *L. salivarius* strains.

The unique genes or the genes sheared by specific strains detected in our comparative genomic analysis are involved in bacterial cell division, in the biosynthesis of the bilayer-forming membrane, the biosynthesis and catabolism of cell-wall peptidoglycan, the biosynthesis of exopolysaccharides (EPS), or are integral inner membrane proteins or involved in the integration of membrane proteins, such as lipoproteins. These results strongly suggest that the cell wall and the surface molecules expressed in the different FFIG strains are involved in their differential capacity to modulate the immune response of PIE cells.

To confirm these findings, we further performed a genomic comparison of the strains with the ability to increase IFN-β, *L. salivarius* FFIG58, and FFIG23, with the non-immunomodulatory strain FFIG124 (Figure S3). The comparative study of these three strains revealed a coregenome of 1521 genes. The strains FFIG58 and FFIG23 had 39 and 59 unique genes, respectively, while they shared 356 genes. *L. salivarius* FFIG124 had 58 unique genes and only sheared 21 genes with the FFIG58.

Among the genes sheared by FFIG58 and FFIG23 strains, we found the inner membrane proteins YdjM (*ydjM*), YdiM (*ydiM*), YbaN (*ybaN*), YbhL (*ybhL*), the glycosyltransferase EpsD (*epsD*), the peptidoglycan O-acetyltransferase (*patA2*), N-acetylglucosaminyldiphosphoundecaprenol N-acetyl-beta-D-mannosaminyltransferase (*tagA*), the teichoic acids export ATP-binding protein (*tagH*) and the glycosyltransferase EpsJ (*epsJ*) (Table S5). Among the unique genes for the FFIG124 strain, we found a UDP-N-acetylglucosamine 2-epimerase (*mnaA*). These results further highlight that the differences in the bacterial surface molecules would determine their ability to modulate immune responses in PIE cells.

The genes *dapX*, *patA1*, *patA3*, and *patA5* are involved in the acetylation of the peptidoglycan. It was shown that N- and O-acetylation of the cell wall peptidoglycan of Gram-positive bacteria are involved in conferring resistance to different types of antimicrobial compounds targeting the cell wall, such as lysozyme, β-lactam antibiotics, endogenous autolysins, and bacteriocins [41,42]. Furthermore, N- and O-acetylation have been shown to differentially modulate the recognition of bacteria by the innate immune system [42]. Importantly, the extent of the modification of peptidoglycan by acetylation in bacterial cells was shown to vary with species, strain, and even culture conditions [42]. The peptidoglycan O-acetyltransferases *patA* are involved in the O-acetylation of the peptidoglycan. Although the function of *patA* genes has been demonstrated in Gram-negative bacteria and streptococci, their function has not been evaluated experimentally in lactobacilli.

The results allow us to speculate that differences in the cell wall, in particular, in the molecular structure of peptidoglycan, could explain at least partially the differential immunomodulatory activity observed in the *L. salivarius* strains isolated from the wakame-fed pigs. In line with this hypothesis, we have previously demonstrated that differences in peptidoglycans can confer lactobacilli strains a different ability to interact with the immune system, and consequently, to differentially modulate immune responses [43,44]. Transcriptomic studies performed in PIE cells [18,38] and macrophages [44] evaluating the innate immune response triggered by TLR3 activation demonstrated that both *L. rhamnosus* CRL1505 and *L. plantarum* CRL1506 were capable to differentially modulate the expression of immune and immune-related genes. When the immunomodulatory effects of viable bacteria were compared with the purified peptidoglycans, only the peptidoglycan from the CRL1505 strain was capable of modulating TLR3-mediated immune response in a similar way as the viable lactobacilli. Interestingly, the ability of *L. plantarum* CRL1506 to modulate TLR3-mediated innate immune in PIE cells was exerted at least partially by its lipoteichoic acid and not its peptidoglycan [45].

**Figure 4:**
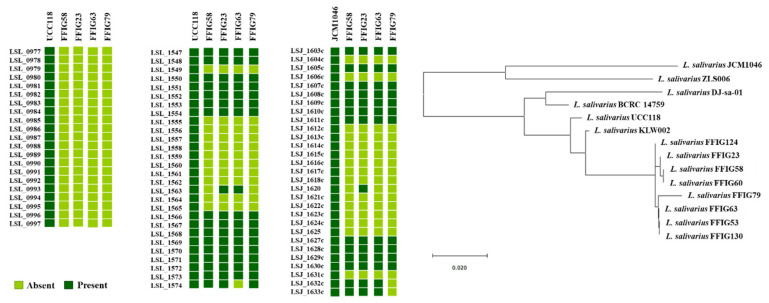
Genomic comparison of the exopolysaccharides (EPS) clusters from *Ligilactobacillus salivarius* UCC118 and JCM1046 and the *L. salivarius* strains isolated from the intestinal mucosa of wakame-fed pigs. The phylogenetic tree was constructed by using the sequences of the twelve genes sheared by the strains that are conserved and were considered as EPS core genes.

In addition, taking into consideration that some of the genes detected to be strain-specific are involved in EPS biosynthesis, we further examined EPS-related genes in the genomes of the *L. salivarius* strains isolated from the intestinal mucosa of wakame-fed pigs. For this purpose, we used as reference the EPS clusters described in the *L. salivarius* strains UCC118 [40] and JCM1046 [46]. Two EPS gene clusters were described in *L. salivarius* UCC118: The EPS cluster 1 containing 20 genes and the EPS cluster 2 containing 27, while one EPS gene cluster was described in *L. salivarius* JCM1046 containing 28 genes (Table S6). The EPS cluster from the JCM1046 strain was designated as EPS cluster 3 in this work for clarity. None of the genes of the EPS cluster 1 were detected in the genomes of the FFIG23, FFIG58, FFIG63, or FFIG79 (Figure 4, Table S6). This is in line with previous genomic studies of Harris et al. [40], who described that the EPS cluster 1 was found mainly in *L. salivarius* strains of human origin, suggesting that this gene cluster might code for an adaptive trait to the human gastrointestinal tract.

On the other hand, the conserved genes in the EPS cluster 2 and 3 [40,46], which include an undecaprenyl-phosphate β-glucosephosphotransferase, a transcriptional regulator LytR, a phosphotyrosine-protein phosphatase, a tyrosine-protein kinase, a chain length regulator, a β-N-acetylhexosaminidase, a dTDP-4-dehydrorhamnose reductase, a dTDP-glucose 4,6-dehydratase, a dTDP-4-dehydrorhamnose 3,5-epimerase, and a glucose-1-phosphate thymidylyltransferase were found in the FFIG strains (Figure 4, Table S6). The gene LSL_1549 (glycosyltransferase) and the genes LSL_1555 to LSL_1565, corresponding mainly to glycosyltransferases and transposases were not found in the genomes of FFIG strains. The only exception was the transposase ISLasa4t (LSL_1563) that was detected in the FFIG23 and FFIG63 strains (Figure 4, Table S6). In addition, the glycosyltransferase LSL_1574 from the EPS cluster 2 was not found in the FFIG63 genome. Similar results were obtained when the EPS cluster 3 was used to analyze the FFIG genomes. These results were expected, since the EPS clusters 2 and 3 had substantial similarities [40,46]. Interestingly, the genes LSJ_1604c (glycosyltransferase), LSJ_1606c (glycosyltransferase), and LSJ_1631c (α-L-Rha α-1,3-L-rhamnosyltransferase) from the EPS cluster 3 were not found in the FFIG genomes. Furthermore, the genes LSJ_1632c and LSJ_1633c corresponding to glycosyltransferases were not detected in the FFIG79, while they were found in the FFIG23, FFIG58, and FFIG63 strains (Figure 4, Table S6).

By analyzing the conserved genes in the EPS clusters 2 and 3 (Figure S4), we defined 12 EPS core genes that were used to further analyze EPS in all the genomes of the *L. salivarius* strains isolated from the intestinal mucosa of wakame-fed pigs. The phylogenetic tree constructed with the sequences of the 12 EPS core genes revealed that all the FFIG strains clustered separately from the other *L. salivarius* strains from the porcine origin, as well as from the strains obtained from human and chicken intestine (Figure 4). Among the FFIGs strains, the most remarkable difference found in the comparison of the 12 EPS core genes was detected for FFIG79 that separated from the group constituted by FFIG53, FFIG63, and FFIG130. Interestingly, all the FFIG strains clustered separately from *L. salivarius* BCRC 14759 (Figure 4), which has been described as a highly EPS-producing strain [47].

**Figure 5:**
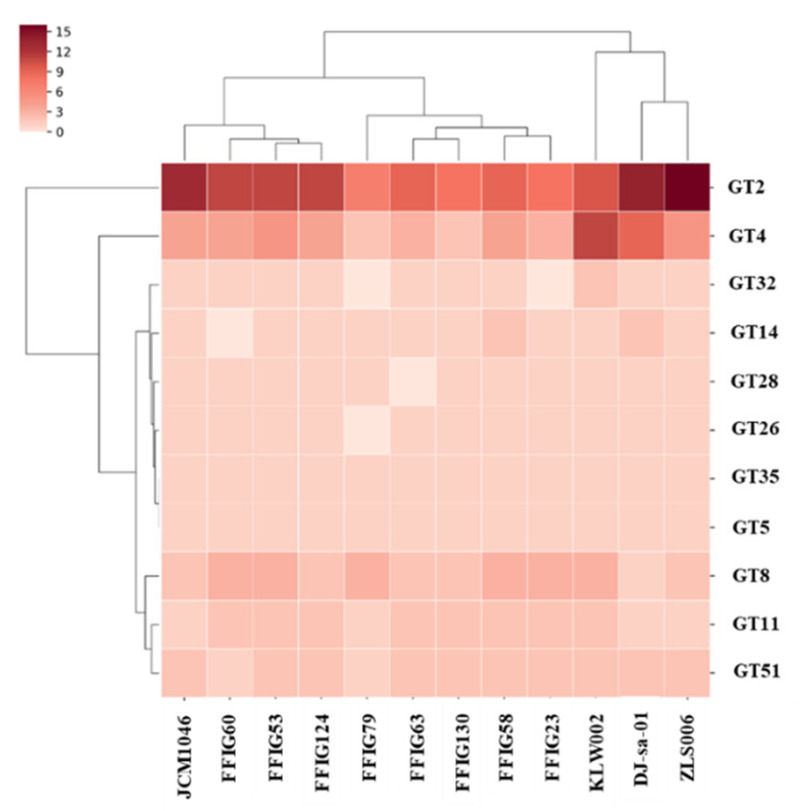
Genomic comparison of the glycosyltransferases present in the genomes of *Ligilactobacillus salivarius* strains isolated from the intestinal mucosa of wakame-fed pigs and other *L. salivarius* strains of porcine origin with available public genomes. The heat-map was constructed considering the numbers of glycosyltransferases in each family.

Although all the FFIG strains contained essentially the same EPS genes, our results evaluating the phylogenetic clustering, based on the nucleotide sequences of 12 conserved genes, showed some degree of divergence. These results are in line with previous genomics studies performed by Lee et al. [11], who used 24 conserved EPS genes to evaluate the differences between *L. salivarius* isolated from humans, pigs, and chickens, and found divergence associated with the hosts. The work suggested that the point mutations in the EPS genes rather than gene acquisition/lost to explain the capability of *L. salivarius* strains to produce different EPS. In addition, it is known that the presence of different types of glycosyltransferases in the genome of EPS-producing bacteria can modify the structure of the EPS molecule, and consequently, its functionality. In this regard, Harris et al. [40] demonstrated a different abundance of genes belonging to glycosyltransferases families among 42 *L. salivarius* strains, and highlighted that the set of glycosyltransferases among each strain could be involved in the generation of variable structures of EPS that would be associated with their different abilities to interact with abiotic and biotic environmental factors. Then, we also evaluated the abundance of genes belonging to glycosyltransferases families among the FFIG strains and compared them with the other four *L. salivarius* strains of porcine origin (Figure 5). Interestingly, the clustering analysis, considering the numbers and types of glycosyltransferases showed similarities between the immunomodulatory strains FFIG23 and FFIG58. In addition, the non-immunomodulatory FFIG124 and the strain FFIG79 clustered separated from the others.

We have demonstrated that the EPS from the immunobiotic *L. delbrueckii* subsp. *Delbrueckii* TUA4408L was able to diminish the activation of NF-kB and MAPK pathways in PIE cells after the activation of TLR4, reducing the expression of the pro-inflammatory cytokines IL-6, IL-8, and MCP-1 [19]. In addition, the EPS of the TUA4408L strains was capable of increasing the activation of the IRF-3 pathway, inducing the expression of IFN-β and antiviral factors in PIE cells and enhancing their resistance to rotavirus infection [17]. Moreover, the immunomodulatory effects induced by the TUA4408L strain in PIE cells in the context of TLR3 activation could be reproduced when using only the acidic purified fraction of its EPS. We also demonstrated that the EPS of *L. delbrueckii* subsp. *Bulgaricus* OLL1037R-1 is capable of improving IFN-β and antiviral factors in PIE cells, but the complete EPS molecule was necessary for obtaining the highest antiviral activity [48]. These previous studies showed that the immunomodulatory properties of the EPS produced by lactobacilli are strain-specific, and therefore, each EPS should be studied in-depth, since it is not possible to make extrapolations even with related strains or strains of the same species.

Thus, it is necessary to carry out more studies to determine the nature of peptidoglycan molecules, to evaluate if the *L. salivarius* FFIG strains are able to produce EPS, and to determine the exact immunomodulatory capacity of each peptidoglycan and EPS. These studies would be of value to determine whether the differences in the peptidoglycan and/or EPS molecules of FFIG strains could explain their differences in the ability to modulate the innate immunity in PIE cells.

### 3.4. Characterization of the Ability of L. salivarius Isolated from the Intestinal Tract of Wakame-Fed Pigs to Adhere to Mucins and PIE Cells

**Figure 6:**
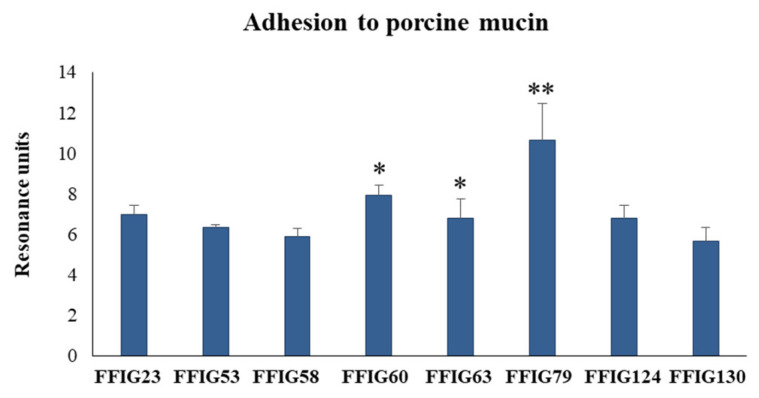
Adhesion of *Ligilactobacillus salivarius* strains isolated from the intestinal mucosa of wakame-fed pigs to porcine mucins. The results represent data from three independent experiments. Asterisks indicate significant differences when compared to the strain with the lowest adhesion ability (* *p* < 0.05, ** *p* < 0.01).

It is considered that one of the most important properties of probiotic lactobacilli is their ability to colonize the gastrointestinal tract of the host in which they are expected to exert their beneficial effects. The colonization ability is related to the capacity of lactobacilli to bind and adhere to mucus and intestinal epithelial cells. Then, we next evaluated the ability of the *L. salivarius* FFIG strains to adhere to porcine mucins. The porcine mucin from the small intestine was purified, as described in the material and methods. The mucin fractions (Figure S5) were then used to evaluate the adhesion ability of *L. salivarius* isolated from the intestinal tract of wakame-fed pigs (Figure 6). All the studied strains had the ability to adhere to porcine mucins, a fact that was expected considering the origin of the FFIG strains. *L. salivarius* FFIG23, FFIG53, FFIG58, FFIG124, and FFIG130 had a modest capacity to adhere to porcine mucins as shown by the resonance units that had values below 6. The FFIG60 and FFIG63 strains had a moderate ability to adhere to porcine mucins (resonance units between 6 and 8), while the FFIG79 had a remarkable capacity to bind and adhere to porcine mucins (resonance units above 8) (Figure 6).

**Figure 7:**
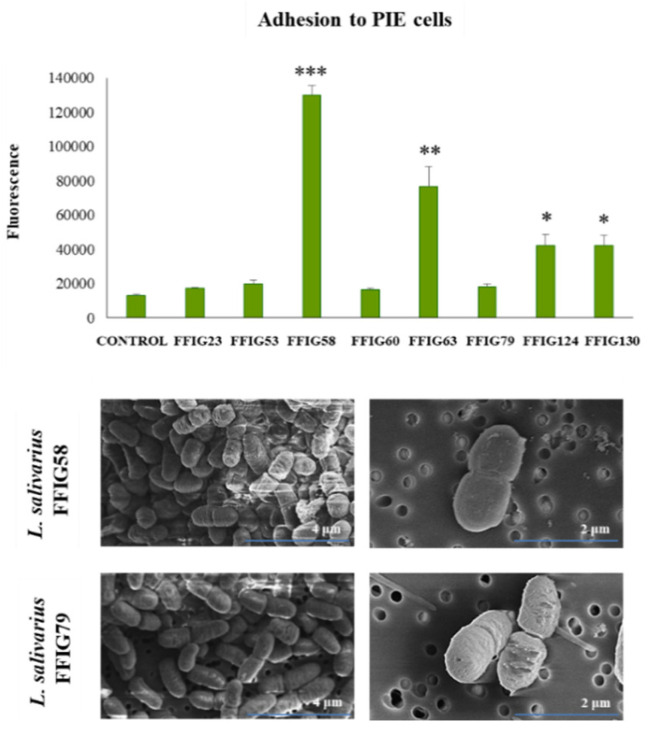
Adhesion of *Ligilactobacillus salivarius* strains isolated from the intestinal mucosa of wakame-fed pigs to porcine intestinal epithelial (PIE) cells. The results represent data from three independent experiments. Asterisks indicate significant differences when compared to the control PIE cells (* *p* < 0.05, ** *p* < 0.01, *** *p* < 0.001). Scanning electron microscope (SEM) analysis of *L. salivarius* FFIG58 and *L. salivarius* FFIG79.

We also observed a strain-dependent effect when the adhesion of lactobacilli to PIE cells was evaluated (Figure 7). *L. salivarius* FFIG23, FFIG53, FFIG60, and FFIG79 could not adhere to PIE cells as shown by the fluorescence units that were not different from control cells. The FFIG124 and FFIG130 strains had a moderate ability to adhere to PIE cells, while the FFIG58 and FFIG63 had a remarkable capacity to bind and adhere to PIE cells (Figure 7); being the adhesion of the FFIG58 strain the most notorious among all the strains evaluated. Taking into consideration the outstanding differences between the FFIG58 and other strains, such as the FFIG79 in their capacity to adhere to PIE cells, we performed an SEM analysis of these two *L. salivarius* strains to evaluate the presence of bacterial structures, such as fimbriae, that may explain their different behavior (Figure 7). No evident differences were found in the SEM analysis when the *L. salivarius* FFIG58 and FFIG79 were compared. These results indicate that at least under these experimental conditions, the FFIG58 strain does not present evident surface structures that may be associated with its greater ability to adhere to porcine epithelial cells.

### 3.5. Comparative Genomic Analysis of L. salivarius Isolated from the Intestinal Tract of Wakame-Fed Pigs Belonging to Different “Adhesion Phenotypes”

**Figure 8:**
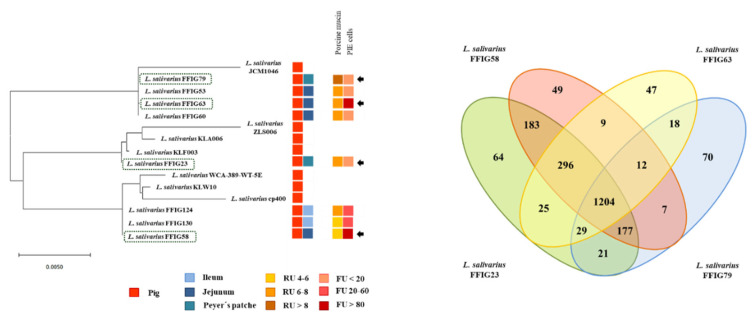
Genomic comparison of *Ligilactobacillus salivarius* strains isolated from the intestinal mucosa of wakame-fed pigs. Four “adhesion phenotypes” were defined according to the ability of *L. salivarius* FFIG strains to adhere to porcine mucins, as well as to porcine intestinal epithelial (PIE) cells. The phylogenetic tree constructed with the MLST analysis of the genes *parB*, *rpsB*, *pheS*, *nrdB*, *groEL*, and *ftsQ* is used to show the strains. *L. salivarius* FFIG23, FFIG58, FFIG63, and FFIG79 were compared. Venn diagram depicts the number of unique genes in each genome and the numbers of genes sheared by the strains.

We next carried out comparative genomic studies to characterize the *L. salivarius* FFIG strains with different adhesion capacities. Thus, we defined “adhesion phenotypes” according to the ability of the FFIG strains to adhere to porcine mucins or to PIE cells (Figure 8). The strain FFIG58 that had a high capacity to adhere to PIE cells and low ability to adhere to mucins, and the strain FFIG79 with the exact opposite behavior were considered as different “adhesion phenotypes”. In addition, the strain FFIG63 with high capacity to adhere to PIE cells and moderate ability to adhere to mucins, and the strain FFIG23 with the moderate capacity to adhere to porcine mucins and low adhesion to PIE cells were considered as two “adhesion phenotypes” groups (Figure 8).

The comparative study of the strains FFIG58, FFIG23, FFIG63, and FFIG79 revealed a coregenome of 1204 genes (Figure 8). The strains with the ability to adhere to PIE cells, *L. salivarius* FFIG58, and FFIG63, had 49 and 47 unique genes, respectively. In addition, the FFIG58 and FFIG63 strains sheared nine genes that were not found in FFIG23 or FFIG79. Among the unique genes of the FFIG58 strain, we found a fap1-like adhesin (*fap1*), while in the genes shared by FFIG58 and FFIG63, we were not able to identify genes potentially involved in adhesion to mucins or intestinal epithelial cells (Table S7). On the other hand, among the unique genes of *L. salivarius* FFIG63, we identified a glycosyltransferase EpsJ (*epsJ*). The strain *L. salivarius* FFIG23 and the FFIG58 sheared 183 genes, including a type 4 prepilin-like proteins leader peptide-processing enzyme or membrane prepilin peptidase (*comC*). In addition, the highly porcine mucin-adhesive strain *L. salivarius* FFIG79 had 64 unique genes, including a putative agglutinin receptor (*ssp5*) that binds sialic acid residues of salivary agglutinin in a calcium-dependent reaction. Among the genes sheared by FFIG23 and FFIG79, we found a lipoteichoic acid synthase 1 (*ltaS1*) (Table S7).

We also performed a comparative genomic study of the strains FFIG58 and FFIG79 (with high adhesion to PIE cells and porcine mucin, respectively) with the less adherent strain FFIG60 (Figure S6). The comparison of these three strains revealed a coregenome of 1362 genes. The strains FFIG58, FFIG79, and FFIG60 had 122, 69, and 53 genes, respectively. Among the unique genes in the FFIG60 strain, we found a glycosyltransferase EpsH (*epsH*) and the lipoteichoic acid synthase 1 (*ltaS1*) (Table S8). The FFIG60 and FFIG79 strains sheared the gene for a glycosyltransferase EpsJ (*epsJ*). Of note, the FFIG60 and FFIG58 sheared 385 genes, and among them, we detected a sugar transferase EpsL (*epsL*), a glycosyltransferase EpsD (*epsD*), and the Fap1-like adhesin (*fap1*) (Table S8).

As we discussed earlier, the cell wall and their associated molecules, as well as the EPS seems to be different in the FFIG strain conferring them different immunomodulatory potential. These two bacterial structures have also been associated with a different adhesion ability of lactobacilli. Earlier studies demonstrated the importance of lipoteichoic acid as a mediator of the adhesion of lactobacilli to human intestinal epithelial cells. Experiments using purified lipoteichoic acid from the probiotic strain *L. johnsonii* La1 showed that preincubation of the Caco-2 cells with increasing amounts of the pure molecule significantly reduced the adhesion of the La1 strain [49]. On the other hand, it was shown that the EPS-producing strain *L. paracasei* BGSJ2-83 is able to adhere to Caco-2 cells, while the non-producing EPS mutant obtained by insertion mutagenesis of the gene encoding a primary glycosyltransferase has a significantly diminished ability to adhere to intestinal cells [50]. In addition, the BGSJ2-83 strains showed a higher adhesion ability than the EPS mutant to HT29-MTX cells, which are able to produce mucins. Studying the role of these two bacterial molecules in the adhesion capacity of FFIG strains to PIE cells and mucins, through the generation of mutants, for example, is an interesting topic for future research.

*L. salivarius* FFIG23 and the FFIG58 sheared a gene for a membrane prepilin peptidase (*comC*) that belongs to the fimbrilin-protein exporter (FPE) system. The FPE pathway is involved in the assembling of the competence pseudo-pili that is part of the cell-surface appendages in Gam-positive bacteria [51]. The competence pseudo-pili is involved in DNA recognition at the cell surface and allows the uptake of exogenous DNA across the bacterial cytoplasmic membrane [52]. The FPE system has been described in several species of lactobacilli [51], but it has not been associated with their adhesion ability to host cells or mucins.

As mentioned above, the *L. salivarius* FFIG79 genome contained a putative agglutinin receptor (*ssp5*) or cell surface agglutinin protein that was not found in the genomes of FFIG23, FFIG58, or FFIG63. We further searched for this gene in the other FFIG strains, and we found it only in the genome of *L. salivarius* FFIG124. Some regions of cell surface agglutinin protein in the FFIG79 and FFIG124 strains show similarities with the ssp5 agglutinin receptor of *Streptococcus gordonii*, the isopeptide-forming adherence proteins from *S. oralis*, the antigen I/II family LPXTG-anchored adhesin of *S. orisasini*, and the salivary agglutinin adherence domain-containing protein from *S. mutants*. However, we only found a maximum of 30% similarity of the putative ssp5 protein in the FFIG strains compared with those found in streptococci. The cell surface agglutinin protein of FFIG79 and FFIG124 strains showed similarities to the antigen I/II family LPXTG-anchored adhesin of *S. orisasini* SH06 in the adhesin P1, the glucan-binding protein C, and the antigen C domains (Figure S7). Blast analysis also revealed a 98.8% of identity (with 68% of query cover) of the cell surface agglutinin protein of FFIG strains with the putative cell surface agglutinin protein from *L. salivarius* cp400, which also contained an antigen C domain (Figure S7). Of note, FFIG79 and FFIG124 strains significantly differed in their abilities to adhere to mucins and PIE cells. While *L. salivarius* FFIG79 was able to adhere to porcine mucin, the FFIG124 strain was more efficient for the adhesion to PIE cells (Figure 6 and Figure 7). Then, it is tempting to speculate that the cell surface agglutinin protein of *L. salivarius* FFIG79 and FFIG124 would not have a key role in the colonization of the intestinal tract. It was reported that the antigen I/II family proteins, including the SSP-5 agglutinin, are capable of binding to sialic acid, fucose, lactose, and N-acetylgalactosamine that are abundant in the mucous glycoproteins present in the human saliva, and therefore, are considered important factors that enable the colonization of bacteria in the oral mucosa [53]. It would be of interest to investigate if strains, such as FFIG79 and FFIG124, are capable of colonizing the porcine oral cavity and if they play a positive role in this mucosa.

Fimbria-associated protein 1 (Fap1) is a glycosylated surface adhesin required for fimbria biogenesis and biofilm formation in *S. parasanguinis*. The secretion of mature Fap1 is dependent on the presence of the accessory system SecA2-SecY2 [54]. Different Fap1-like proteins have been described in several bacterial species [55]. In fact, Fap1-like gene clusters and related glycosylation and secretion loci are present in the genomes of oral streptococci, such as *S. gordonii*, *S. sanguinis*, and *S. crispatus,* as well as in the commensal bacteria *L. johnsonii* and *S. salivarius* [55]. These Fap1-like serine-rich proteins have been shown to belong to an expanding family of adhesins known as serine-rich repeats containing adhesins or srr adhesins [56]. In the comparison of FFIG58, FFIG23, FFIG63, and FFIG79, we found a Fap1-like protein (or a srr adhesin) only in the genome of the FFIG58 strain (Table S7). However, further analysis also revealed the presence of variants of srr adhesins in the genomes of all the remaining FFIG strains (discussed below).

Since the comparative analysis of the genomes of *L. salivarius* isolated from the intestinal tract of wakame-fed pigs did not allow us to identify unique factors that explain their different ability to adhere to mucins or epithelial cells, we further investigated several proteins and systems that were described to be involved in the adhesion of lactobacilli.

### 3.6. Analysis of Adhesion Factors in L. salivarius Isolated from the Intestinal Tract of Wakame-Fed Pigs

The SecA2/SecY2 secretion system has been associated with the ability of some lactobacilli strains to adhere to mucosal tissues [57,58]. The SecA2/SecY2 genomic cluster encodes the motor protein SecA2, the membrane translocation complex SecY2, the chaperones Asp1-3, and the glycosyltransferases Gtf A and B [59,60]. This system facilitates the glycosylation of srr proteins and the exportation of the glycosylated adhesins that are involved in cell adhesion to the host surface [56,61]. The SecA2/SecY2 secretion system was found in *L. reuteri* strains from murine origins, but not in strains isolated from humans [62,63]. Interestingly, the SecA2/SecY2 cluster in the murine *L. reuteri* 100-23 strain was shown to be important for the adhesion of lactobacilli to the forestomach epithelium of mice. In fact, the mutant *L. reuteri* 100-23 strains lacking the *secA2* or the *srr* genes had altered adhesion and reduced gastric colonization ability when compared to the wild-type strain [57]. More recently, it was reported that the SecA2/SecY2 secretion system is involved in the surface expression of two large srr adhesion proteins in the human isolate *L. casei* AMBR2, which facilitates its adhesion to the nasal epithelium [58,64].

**Figure 9:**
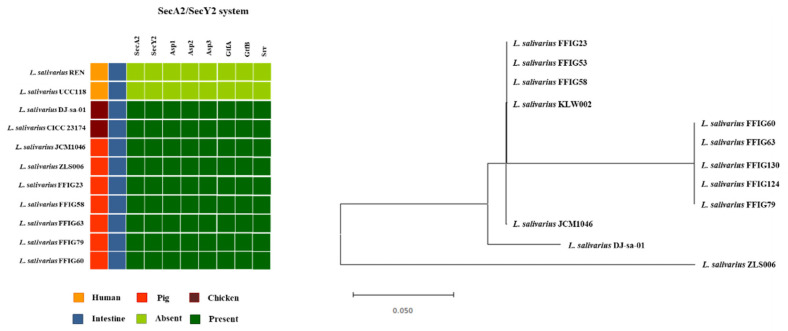
Genomic comparison of the SeA2-SecY2 accessory secretion system from *Ligilactobacillus salivarius* strains isolated from the intestinal mucosa of wakame-fed pigs and probiotic *L. salivarius* strains isolated from the intestinal tract of humans, pigs, and chickens. The phylogenetic tree was constructed by using the sequences of the genes *secA2*, *secY2*, *asp1*, *asp2*, *asp3*, *gtfA*, and *gtfB* sheared by the strains of animal origin.

Of note, it was reported that *L. salivarius* strains isolated from pigs and chickens possess the SecA2-SecY2 cluster, while this system was not detected the human *L. salivarius* isolates [11]. In agreement with this previous finding, we were not able to detect the SecA2-SecY2 cluster in the human-related *L. salivarius* strains UCC118 and REN, which we used as references in this work. Moreover, the SecA2-SecY2 cluster was found in all the genomes of *L. salivarius* strains isolated from the intestine of wakame-fed pigs (Figure 9).

Although all the FFIG strains contained the genes of the SecA2-SecY2 system, our results evaluating the phylogenetic clustering, based on the nucleotide sequences of the conserved genes *secA2*, *secY2*, *asp1*, *asp2*, *asp3*, *gtfA*, and *gtfB*, showed some degree of divergence (Figure 9). The FFIG23, FFIG53, and FFIG58 clustered separately from the other *L. salivarius* strains isolated from the intestine of wakame-fed pigs and together with the porcine strain KLW002. No differences were detected between the FFIG60, FFIG63, FFIG79, FFIG124, and FFIG130. Of note, all the FFIG strain clustered separately from the porcine strains JCM1046 and ZLS006 and the chicken strain DJ-sa-01 (Figure 9).

**Figure 10:**
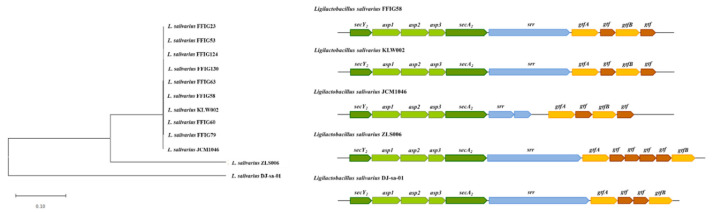
Genomic comparison of the SeA2-SecY2 accessory secretion system from *Ligilactobacillus salivarius* strains isolated from the intestinal mucosa of wakame-fed pigs and probiotic *L. salivarius* strains isolated from the intestinal tract of pigs, and chickens. The phylogenetic tree was constructed by using the sequences of the *srr* genes belonging to the SeA2-SecY2 cluster. Gene organization within the SeA2-SecY2 accessory secretion system for selected strains is shown. Conserved glycosyltransferases are shown in yellow, while non-conserved glycosyltransferases are shown in brown.

We further analyzed the srr proteins located within the SecA2-SecY2 system cluster in all the FFIG strains to detect potential differences between them (Figure 10). The srr protein found in the genome of *L. salivarius* FFIG58 (detected as a fap1-like adhesin in Figure 8) was identical to the srr protein found in the KLW002 genome and slightly different from the srr proteins located in the SecA2-SecY2 system cluster of the other FFIG strains. However, the only difference found between the distinct srr proteins among the FFIG strains was in their length, while the similarity between them was 97–99%. Furthermore, the phylogenetic clustering, based on their nucleotide sequences, revealed no significant divergence (Figure 10). Interestingly, the FFIG strains clustered together with the porcine strain JCM1046 when the srr proteins were compared despite the large difference in the size in their genes. In contrast, evident differences were found when the srr proteins were compared with the porcine strain ZLS006 and the chicken strain DJ-sa-01 (Figure 10).

When the functional domains in the srr proteins of FFIG strains were analyzed, we were able to identify the KxYKxGKxW signal peptide and the serine-rich repeat adhesion glycoprotein AST domain in the N-terminal end, the LPXTG cell wall-anchoring motif at the C-terminus, as well as highly repeated serines in the middle of the molecule (data are not shown). Those sequences have been identified as conserved in the organization of srr proteins, particularly in the adhesins of oral streptococci [56]. However, our analysis did not reveal any known adhesin-associated binding domain the srr proteins of FFIG strains. Our results are in line with the previous genomic analysis performed by Lee et al. [11], who were not able to find srr proteins in the genome of *L. salivarius* of porcine or chicken origins that satisfice all the necessary requirements to consider those proteins as functional adhesins related to the SecA2-SecY2 system. Further studies evaluating the functional properties of the srr proteins and the SecA2-SecY2 system genes in the FFIG strains in the context of adhesion to mucins and PIE cells would be of value to determine their precise role, or the lack of it, in the adhesion capabilities of the *L. salivarius* strains isolated from the intestine of wakame-fed pigs.

Several mucus-binding proteins (MucBPs) have been identified in lactic acid bacteria and were associated with their ability to colonize the gastrointestinal tract [65]. MucBPs contain variable numbers of mub repeats, each of them is divided into two domains, a mucin-binding domain and an immunoglobulin-binding domain [66,67]. These mub repeats are capable of mediating the adhesion of lactobacilli to mucin glycans through interactions with terminal sialic acid [68,69]. Up-to seven different MucBP orthologous were found in the pangenome of *L. salivarius* [11].

**Figure 11:**
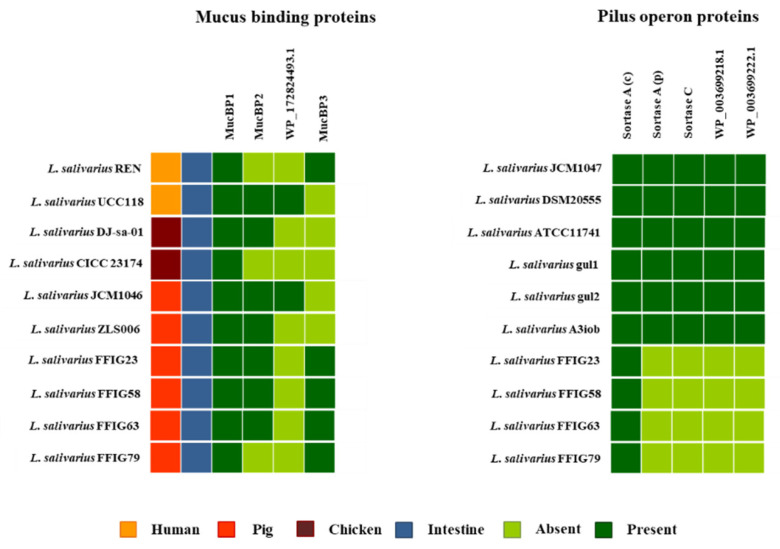
Genomic comparison of the mucus binding proteins and the pilus operon from *Ligilactobacillus salivarius* strains isolated from the intestinal mucosa of wakame-fed pigs with *L. salivarius* strains with available public genomes. Mucus binding proteins were compared with probiotic *L. salivarius* strains isolated from the intestinal tract of humans, pigs, and chickens. Gene of proteins associated with pilus operon were compared to the six *L. salivarius* strains that were predicted to harbor a pilus operon by genomic analysis.

We searched for MucBPs genes in the genomes of *L. salivarius* strains isolated from the intestine of wakame-fed pigs focusing our attention in the “adhesion phenotypes” groups, which included the FFIG23, FFIG58, FFIG63, and FFIG79 strains. In addition, we searched for MucBPs genes in the genomes of the strains UCC118, REN, DJ-sa-01, CICC23174, JCM1046, and ZLS006 for comparison (Figure 11).

As described previously [11], a common MucBP (designated here as MucBP1) was found in all the *L. salivarius* strains, independently of the host origin. The MucBP WP_087118522.1 (designated here as MucBP2; Figure S8) was found in the genomes of the UCC118, DJ-sa-01, JCM1046, and ZLS006, as well as in the strains FFIG23, FFIG58, and FFIG63, but not in the genome of FFIG79. In addition, a MucBP WP_179219866.1 (designated here as MucBP3; Figure S8) was sheared by the REN strain and the *L. salivarius* FFIG strains, while the protein WP_172824493.1 detected in the genomes of UCC118 and JCM1046 were not found in the FFIG strains (Figure 11). MucBP1, were also found in the genomes of the other FFIG strains. MucBP2 was detected in the genomes of FFIG60 and FFIG124, but not in FFIG53 and FFIG130 strains, while MucBP3 was present in FFIG130, but not in FFIG53, FFIG60, and FFIG124 (data are not shown). Our analysis was not able to identify a clear association between the presence of MucBPs and the different adhesion capabilities of the FFIG strains.

Pili are other bacterial structures involved in the intestinal colonization of lactobacilli, which have also been associated with probiotic effects [70]. The pilus is comprised of two to three types of pilin subunits, each of them with its own distinct location and role in the molecular structure. In its biosynthesis, a pilus-specific sortase type C catalyzes the head-to-tail assembly of the pilins into the final polymerized form [71]. Despite the fact that the presence of pilus in probiotic strains belonging to the species *L. salivarius* has been scarcely evaluated, this bacterial structure is predicted to exist in this species based on genomics analyses. In fact, genomic studies performed by Harris et al. [40] showed that among 43 *L. salivarius* genomes, only five contained the genes for an extra sortase A, a sortase C, and putative pilin subunits. These strains are *L. salivarius* JCM1047, DSM20555, ATCC11741, gul1, and gul2. We also searched for other *L. salivarius* strains with pili operon in the NCBI genome bank and found this cluster only in the genome of *L. salivarius* A3iob originally isolated from the bee intestine [24]. Moreover, the comparative analysis of the pili operon sequences in these six strains showed that they harbor only three different pili operons (data are not shown). Then, we used the pili operons of JCM1047, DSM20555, and the A3iob for the genomic comparisons with the *L. salivarius* FFIG23, FFIG58, FFIG63, and FFIG79 (Figure 11; Table S9). No genes for an extra sortase A, a sortase C, or pilin subunits were found in these four strains (Figure 11) or in the other *L. salivarius* strains isolated from the intestine of wakame-fed pigs (data are not shown).

**Figure 12:**
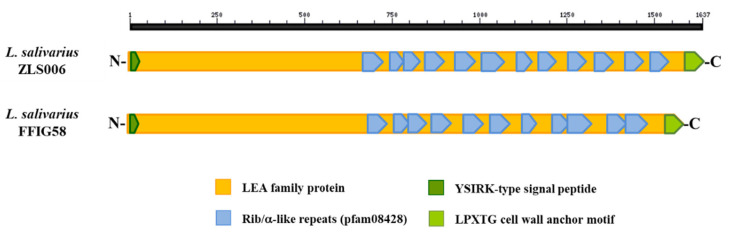
Comparison of the domain organization in the LEA family adhesin from *Ligilactobacillus salivarius* FFIG58 isolated from the intestinal mucosa of wakame-fed pigs and the porcine strain *L. salivarius* ZLS006.

We also investigated the presence of the *Lactobacillus* epithelium adhesin (LEA) family of proteins in the genomes of *L. salivarius* strains. The LEA protein was first described in *L. crispatus* ST1, which is an efficient colonizer of chicken intestines and has the capability to adhere to the stratified squamous epithelial cells of the chicken crop [72]. The work described that the LEA protein differed from other sortase-dependent adhesins from lactobacilli, since it harbored no mub repeats, but instead a highly repeated internal region containing Rib/α-like repeats were present in this protein [72]. The complete genome sequence of the ST1 strain allowed the final characterization of the LEA protein of 1898 amino acids, which contains an N-terminal YSIRK signal sequence, the highly repetitive region of Rib/α-like repeats, and a C-terminal LPxTG anchoring motif [73]. The genomic analysis of the FFIG strains revealed the presence of a similar protein annotated as LEA family epithelial adhesin. The LEA family adhesin from *L. salivarius* FFIG58 is a protein of 1578 amino acids, and contains an N-terminal YSIRK signal sequence, a C-terminal LPxTG anchoring motif, and 11 Rib/α-like repeats (Figure 12). The LEA proteins in all the *L. salivarius* strains isolated from the intestine of wakame-fed pigs were identical (data are not shown), and they were also similar to the LEA proteins found in the genome of *L. salivarius* strains of porcine origin. In fact, the LEA family adhesin from the porcine strain *L. salivarius* ZLS006 has the YSIRK and LPxTG motifs together with 12 Rib/α-like repeats (Figure 12). The LEA family adhesins from FFIG58 and ZLS006 were 90.61% identical.

Of note, proteins with Rib/α-like repeats in lactobacilli have been associated with the binding to the stratified squamous epithelial cells, and consequently, they are considered adhesin facilitating the binding to vaginal epithelial cells [72,74]. A recent study evaluating the temporal variations of gut-associated microbiota in piglets in the first month after birth demonstrated that the small and large intestines sheared a similar composition profile at birth, and while the small intestinal microbiota remained relatively stable, the large intestine quickly expanded and diversified by day 35 [3]. Interestingly, the work revealed that the microorganisms from the maternal milk were the main colonizers of the small intestine (approximately 90%), and although the bacteria of maternal milk contributed to 90% of the microbiota in the large intestine after birth, their presence gradually diminished, and they were replaced by fecal microbes derived from mothers by day 35. Then, vertically transmitted maternal milk and intestinal microorganisms would be of key importance in the strengthening of intestinal barrier functions and the development of the mucosal immune system in porcine neonates. Of note, the remaining 10% of the microbial population that initially colonizes the gut of piglets comes from the vagina and the areolar skin of mothers, which also contain diverse bacterial communities. Although the vaginal seeding is transient after the piglet’s birth, it was suggested that this initial microbial acquisition from the mother is involved in the preparation of the porcine newborns for host-microbial symbiosis [3] as it was also suggested for mouse [75] and human [76] neonates. Then, considering that lactobacilli species have been isolated not only from the gastrointestinal tract of pigs, but also from porcine vaginal mucosa and maternal milk [77,78], the presence of the LEA family adhesins in the genome of FFIG strains raise the question of whether these bacteria can also be found in other ecological niches like the vaginal mucosa. The presence of these immunomodulatory bacteria in the various maternal niches could be a mechanism to ensure the colonization of the newborn’s gastrointestinal tract by microorganisms that help the immune system to mature. In support of this hypothesis, it was shown that that porcine maternally transmitted bacteria had a strong correlation with the expression of antimicrobial peptides, pattern recognition receptors (PRRs), and immunoregulatory cytokines in the gut, highlighting the involvement of maternally derived microorganism in the maturation of the intestinal immune system [3].

### 3.7. L. salivarius FFIG58 Increase the Resistance of PIE Cell to Rotavirus Infection

**Figure 13:**
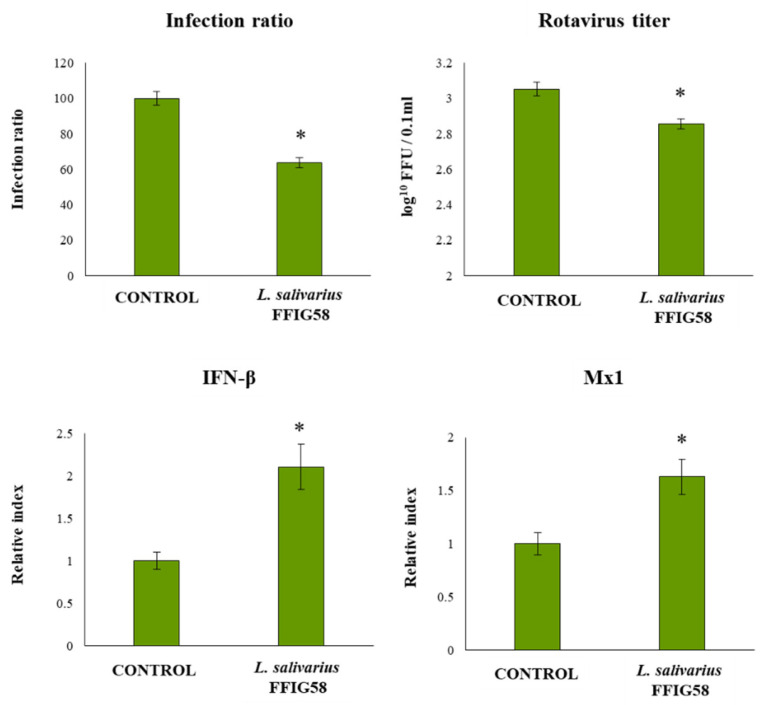
Effect of *Ligilactobacillus salivarius* FFIG58 on the resistance of porcine intestinal epithelial (PIE) cells to rotavirus infection. PIE cells were stimulated with the FFIG58 strain isolated from wakame-fed pigs then challenged with rotavirus. PIE cells with no lactobacilli treatment and challenged with rotavirus were used for comparisons. Rotavirus infection was evaluated by immunofluorescence assay. The protective ability of lactobacilli was examined by calculating the virus titer and the infection ratio. The results represent data from three independent experiments. Asterisks indicate significant differences when compared to the rotavirus control group (* *p* < 0.05).

Finally, we aimed to evaluate whether *L. salivarius* FFIG58, the strain with the most remarkable ability to adhere to PIE cells and modulate innate immunity, was able to confer protection to intestinal cells against a real infectious challenge. Considering the ability of the FFIG58 strain to improve IFN-β and Mx1, we selected rotavirus for our challenge experiments. Then, PIE cells were treated with *L. salivarius* FFIG58 and subsequently challenged with rotavirus (Figure 13). As we reported previously [36], PIE cells are susceptible to rotavirus infection. Of note, the FFIG58 strain was capable of significantly reducing the rotavirus titers, as well as infection ratio in challenged PIE cells. The reduction of rotavirus replication induced by *L. salivarius* FFIG58 was associated with significant improvements in the expressions of IFN-β and Mx1 after the viral challenge (Figure 13).

## 4. Conclusions

**Figure 14:**
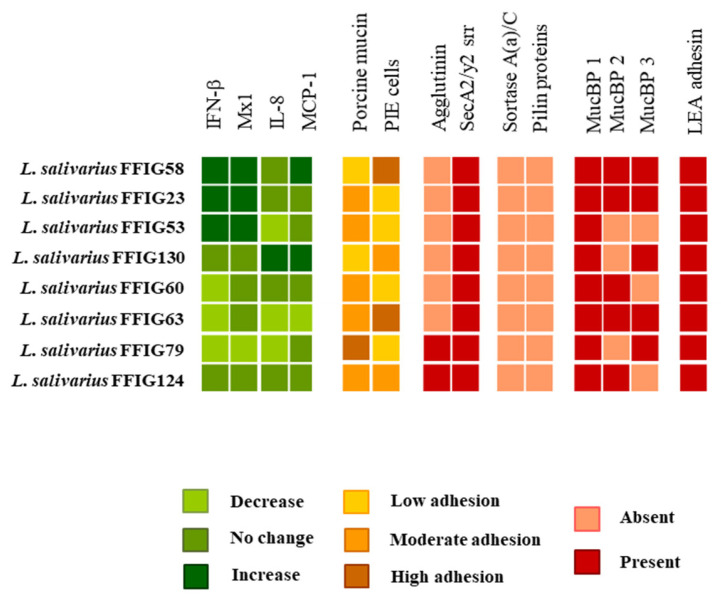
Comparison of the immunomodulatory and adhesion capabilities of *Ligilactobacillus salivarius* isolated from the intestinal mucosa of wakame-fed pigs.

In the present work, we carried out a functional and genomic characterization of several *L. salivarius* strains isolated from the gastrointestinal tract of wakame-fed pigs and found that their immunomodulatory and adhesion capabilities are a strain-dependent characteristic (Figure 14). Our analysis indicated that the differential immunomodulatory activities of FFIG strains in the context of innate immune responses triggered by the activation of TLR3 or TLR4 in PIE cells would be dependent mainly on bacterial surface structures, such as peptidoglycan and EPS. The combination of structural and biochemical variations of these two molecules could be differentially detected by PRRs expressed in epithelial cells, inducing the activation of different signaling pathways, which would translate into a different modulation of the innate immune responses. Further studies using purified molecules or mutant strains would be of great importance to clarify the exact role of peptidoglycan and EPS in the immunomodulatory effects induced by the FFIG strains.

Our results also showed a different adhesion capacity to porcine mucins and PIE cells for each of the FFIG strains. We were not able to identify any particular molecule or group of molecules that could explain the different adhesion capabilities of the *L. salivarius* strains isolated from the intestine of wakame-fed pigs. It is tempting to speculate that in the same way that occurs with the immunomodulatory activity, the different adhesive capacity of the FFIG strains depends on the combination of several factors acting simultaneously and that have been described to be involved in the adhesion of lactobacilli to the intestinal tract, such as EPS, lipoteichoic acid, MucBP, and other adhesins.

Of note, the adhesion to the intestinal mucosa is considered an important trait for bacterial retention in the gastrointestinal tract and for the induction of immunomodulatory effects. The results presented in this work contradict these previous statements. In our hands, the three *L. salivarius* strains (FFIG23, FFIG53, and FFIG58) capable of efficiently increasing the expression of IFN-β and Mx1 in PIE cells showed different capacities to bind to mucins and PIE cells (Figure 14). In addition, the two strains with the highest capacity to bind to PIE cells (FFIG58 and FFIG63) did not modulate in the same way the response of epithelial cells to the challenge with the TLR3 ligand. While *L. salivarius* FFIG58 showed a remarkable ability to increase IFN-β and Mx1 expression levels, the FFIG63 strain did not modify Mx1 levels and even decreased IFN-β values (Figure 14). Another example is given by the group comprised of the FFIG79, FFIG23, and FFIG53 strains, which all presented a low adhesion to PIE cells. However, while FFIG53 and FFIG23 strains increased the expression of IFN-β and Mx1, *L. salivarius* FFIG79 decreased the values of both factors (Figure 14). Then, our results indicate that there is no correlation between the immunomodulatory capacity and the adhesion ability to mucin and epithelial cells. Therefore, in the selection of strains destined to colonize the intestinal mucosa and modulate the immunity of the host, both properties must be adequately evaluated. Interestingly, we showed that *L. salivarius* FFIG58 functionally modulated the innate immune responses triggered by TLR3 and TLR4 activation in PIE cells and efficiently adhered to these cells. Moreover, the FFIG58 strain was able to increase the resistance of PIE cells to the challenge with rotavirus. Therefore, *L. salivarius* FFIG58 is a good candidate for further in vivo studying the protective effect of lactobacilli against intestinal infections in the porcine host.

We hypothesized that a synergistic combination of the immunomodulatory effects of wakame [16] and selected *L. salivarius* strains isolated from the intestine of wakame-fed pigs could be used as a highly efficient functional feed to improve immune health status and reduce the severity of intestinal infections in weaned piglets. In addition to possess immunomodulatory and adhesion activities, *L. salivarius* strains selected for the development of this kind of functional feed should have the ability to metabolize and growing in wakame by-products like stalk and root [15]. In this work, we reported and analyzed, for the first time, the complete genome of several *L. salivarius* strains that were isolated from the intestine of pigs after the selective pressure of feeding the animals with wakame. Further genomic analysis could be of value to reveal the metabolic characteristics and potential of the FFIG strains in general and in the FFIG58 strain, in particular, relating to wakame by-products assimilation, as well as sugar assimilation and synthesis.

## Figures and Tables

**Table 1:** Comparison of the general genome features of sequenced *Ligilactobacillus salivarius* strains isolated from the intestinal mucosa of wakame-fed pigs. *L. salivarius* genomes are available in the NCBI database, and the accession numbers are provided.

*Ligilactobacillus salivarius* Strain	Host	Sample	Genome Size (bp)	G+C Content (%)	Protein-Coding Genes	GenBank ID	Reference
FFIG58	*Sus scrofa*	Intestine	1,984,018	32.8	1891	JACBJR000000000.1	[20]
FFIG23	*Sus scrofa*	Intestine	2,041,027	32.8	1932	JACBJS000000000.1	This work
FFIG53	*Sus scrofa*	Intestine	1,948,231	32.9	1863	JACBJT000000000.1	This work
FFIG60	*Sus scrofa*	Intestine	1,948,639	33.0	1855	JACBJU000000000.1	This work
FFIG63	*Sus scrofa*	Intestine	1,777,024	33.3	1786	JACBJV000000000.1	This work
FFIG79	*Sus scrofa*	Intestine	1,718,597	33.4	1728	JACBJW000000000.1	This work
FFIG124	*Sus scrofa*	Intestine	1,806,583	33.2	1812	JACBJX000000000.1	This work
FFIG130	*Sus scrofa*	Intestine	1,862,635	33.1	1778	JACBJY000000000.1	This work

## Supplementary Materials

The following are available online at https://www.mdpi.com/2076-2607/8/11/1659/s1. Table S1. *Ligilactobacillus salivarius* strains were evaluated in this study. Table S2. General genomic features of *Ligilactobacillus salivarius* strains isolated from the intestinal tract of wakame-fed pigs. Table S3. Comparison of the general genome features of sequenced *Ligilactobacillus salivarius* strains with available public genomes. *L. salivarius* genomes were obtained from the NCBI database. Table S4. Genomic comparison of *Ligilactobacillus salivarius* strains isolated from the intestinal mucosa of wakame-fed pigs. Four “immune phenotypes” were defined according to the ability of *L. salivarius* FFIG strains of modulating toll-like receptors (TLRs) mediated innate immune responses in porcine intestinal epithelial (PIE) cells. *L. salivarius* FFIG23, FFIG58, FFIG79, and FFIG130 were compared. Table S5. Genomic comparison of *Ligilactobacillus salivarius* strains isolated from the intestinal mucosa of wakame-fed pigs. Four “immune phenotypes” were defined according to the ability of *L. salivarius* FFIG strains of modulating (TLRs) mediated innate immune responses in porcine intestinal epithelial (PIE) cells. *L. salivarius* FFIG23, FFIG58, and FFIG124 were compared. Table S6. Genomic comparison of *Ligilactobacillus salivarius* strains isolated from the intestinal mucosa of wakame-fed pigs with probiotic *L. salivarius* UCC118 and JCM1046. The exopolysaccharides genes of the UCC118 and JCM1046 strains were searched in the genomes of FFIG strains. Table S7. Genomic comparison of *Ligilactobacillus salivarius* strains isolated from the intestinal mucosa of wakame-fed pigs. “Adhesion phenotypes” were defined according to the ability of *L. salivarius* FFIG strains to adhere to porcine mucins, as well as to porcine intestinal epithelial (PIE) cells. Table S8. Genomic comparison of *Ligilactobacillus salivarius* strains isolated from the intestinal mucosa of wakame-fed pigs. Three “adhesion phenotypes” are defined according to the ability of *L. salivarius* FFIG strains to adhere to porcine mucins and to porcine intestinal epithelial (PIE) cells. *L. salivarius* FFIG58, FFIG60, and FFIG79 were compared. Table S9. Genomic comparison of proteins associated with pilus operon from *Ligilactobacillus salivarius* strains isolated from the intestinal mucosa of wakame-fed pigs and *L. salivarius* strains harbor pili structures. Figure S1. Clustering of pair-wise average nucleotide identity (ANI) scores of *Ligilactobacillus salivarius* strains isolated from the intestinal mucosa of wakame-fed pigs and compared with *L. salivarius* strains with available public complete genomes. Figure S2. Hierarchical clustering of *Ligilactobacillus salivarius* strains isolated from the intestinal mucosa of wakame-fed pigs. The phylogenetic trees were constructed based on the 16s RNA extracted from the genomes of FFIG strains, as well as from *L. salivarius* strains with available public complete genomes. Figure S3. Genomic comparison of *Ligilactobacillus salivarius* FFIG58, FFIG23, and FFIG124 isolated from the intestinal mucosa of wakame-fed pigs. Venn diagram depicts the number of unique genes in each genome and the numbers of genes sheared by the strains. Figure S4. Comparison of the exopolysaccharide (EPS) clusters from *Ligilactobacillus salivarius* UCC118 and JCM1046. The genes sheared by the two strains (grey) are conserved and were considered as EPS core genes. Dotted lines indicate non-functional genes. Figure S5. Purification of human and porcine mucins to evaluate the adhesion of *Ligilactobacillus salivarius* strains isolated from the intestinal mucosa of wakame-fed pigs. Figure S6. Genomic comparison of *Ligilactobacillus salivarius* strains isolated from the intestinal mucosa of wakame-fed pigs. Three “adhesion phenotypes” defined according to the ability of *L. salivarius* FFIG strains to adhere to porcine mucins and to porcine intestinal epithelial (PIE) cells were compared. *L. salivarius* FFIG58, FFIG60, and FFIG79. Venn diagram depicts the number of unique genes in each genome and the numbers of genes sheared by the strains. Figure S7. Comparison of the putative cell surface agglutinin proteins from *Ligilactobacillus salivarius* FFIG124 and cp400 with the antigen I/II family LPXTG-anchored adhesin from *Streptococcus orisanini* SH06. The antigen I/II family LPXTG-anchored adhesin is a protein of 1,518 amino acids, while the putative cell surface agglutinin proteins from the FFIG124 and cp400 strains are proteins of 1949 and 1420 amino acids, respectively. Figure S8. Comparison of the domain organization of mucus binding proteins (MucBP) from *Ligilactobacillus salivarius* strains isolated from the intestinal mucosa of wakame-fed pigs.

## References

[1] Han G.G., Lee J.Y., Jin G.D., Park J., Choi Y.H., Kang S.K., Chae B.J., Kim E.B., Choi Y.J. (2018). Tracing of the fecal microbiota of commercial pigs at five growth stages from birth to shipment. Sci. Rep..

[2] Crespo-Piazuelo D., Estellé J., Revilla M., Criado-Mesas L., Ramayo-Caldas Y., Óvilo C., Fernández A.I., Ballester M., Folch J.M. (2018). Characterization of bacterial microbiota compositions along the intestinal tract in pigs and their interactions and functions. Sci. Rep..

[3] Liu H., Zeng X., Zhang G., Hou C., Li N., Yu H., Shang L., Zhang X., Trevisi P., Yang F. (2019). Maternal milk and fecal microbes guide the spatiotemporal development of mucosa-associated microbiota and barrier function in the porcine neonatal gut. BMC Biol..

[4] Suda Y., Villena J., Takahashi Y., Hosoya S., Tomosada Y., Tsukida K., Shimazu T., Aso H., Tohno M., Ishida M. (2014). Immunobiotic Lactobacillus jensenii as immune-health promoting factor to improve growth performance and productivity in post-weaning pigs. BMC Immunol..

[5] Liao S.F., Nyachoti M. (2017). Using probiotics to improve swine gut health and nutrient utilization. Anim. Nutr..

[6] Reyer H., Oster M., McCormack U.M., Muráni E., Gardiner G.E., Ponsuksili S., Lawlor P.G., Wimmers K. (2020). Host-microbiota interactions in ileum and caecum of pigs divergent in feed efficiency contribute to nutrient utilization. Microorganisms.

[7] Villena J., Kitazawa H. (2014). Modulation of intestinal TLR4-inflammatory signaling pathways by probiotic microorganisms: Lessons learned from Lactobacillus jensenii TL2937. Front. Immunol..

[8] Wang W., Gänzle M. (2019). Toward rational selection criteria for selection of probiotics in pigs. Adv. Appl. Microbiol..

[9] Aluthge N.D., van Sambeek D.M., Carney-Hinkle E.E., Li Y.S., Fernando S.C., Burkey T.E. (2019). Board Invited review: The pig microbiota and the potential for harnessing the power of the microbiome to improve growth and health. J. Anim. Sci..

[10] Zheng J., Wittouck S., Salvetti E., Franz C.M.A.P., Harris H.M.B., Mattarelli P., O’Toole P.W., Pot B., Vandamme P., Walter J. (2020). A taxonomic note on the genus Lactobacillus: Description of 23 novel genera, emended description of the genus Lactobacillus Beijerinck 1901, and union of Lactobacillaceae and Leuconostocaceae. Int. J. Syst. Evol. Microbiol..

[11] Lee J.Y., Han G.G., Kim E.B., Choi Y.J. (2017). Comparative genomics of *Lactobacillus salivarius* strains focusing on their host adaptation. Microbiol. Res..

[12] Zhang J., Deng J., Wang Z., Che C., Li Y.F., Yang Q. (2011). Modulatory effects of *Lactobacillus salivarius* on intestinal mucosal immunity of piglets. Curr. Microbiol..

[13] Lo Verso L., Lessard M., Talbot G., Fernandez B., Fliss I. (2018). Isolation and Selection of Potential Probiotic Bacteria from the Pig Gastrointestinal Tract. Probiotics Antimicrob. Proteins.

[14] Yoshinaga T., Nishiduka H., Nanba N. (2014). Genotype analysis of commercial products of the soft seaweed Undaria pinnatifida (Laminariales, Alariaceae). Coast. Mar. Sci..

[15] Masumizu Y., Zhou B., Kober A.K.M.H., Islam M.A., Iida H., Ikeda-Ohtsubo W., Suda Y., Albarracin L., Nochi T., Aso H. (2019). Isolation and immunocharacterization of *Lactobacillus salivarius* from the intestine of wakame-fed pigs to develop novel “immunosynbiotics”. Microorganisms.

[16] Katayama M., Fukuda T., Okamura T., Suzuki E., Tamura K., Shimizu Y., Suda Y., Suzuki K. (2011). Effect of dietary addition of seaweed and licorice on the immune performance of pigs. Anim. Sci. J..

[17] Kanmani P., Albarracin L., Kobayashi H., Hebert E.M., Saavedra L., Komatsu R., Gatica B., Miyazaki A., Ikeda-Ohtsubo W., Suda Y. (2018). Genomic characterization of Lactobacillus delbrueckii TUA4408L and evaluation of the antiviral activities of its extracellular polysaccharides in porcine intestinal epithelial cells. Front. Immunol..

[18] Albarracin L., Kobayashi H., Iida H., Sato N., Nochi T., Aso H., Salva S., Alvarez S., Kitazawa H., Villena J. (2017). Transcriptomic analysis of the innate antiviral immune response in porcine intestinal epithelial cells: Influence of immunobiotic lactobacilli. Front. Immunol..

[19] Wachi S., Kanmani P., Tomosada Y., Kobayashi H., Yuri T., Egusa S., Shimazu T., Suda Y., Aso H., Sugawara M. (2014). Lactobacillus delbrueckii TUA4408L and its extracellular polysaccharides attenuate enterotoxigenic *Escherichia coli*- induced inflammatory response in porcine intestinal epitheliocytes via Toll-like receptor-2 and 4. Mol. Nutr. Food Res..

[20] Huang I.-N., Okawara T., Watanabe M., Kawai Y., Kitazawa H., Ohnuma S., Shibata C., Horii A., Kimura K., Taketomo N. (2013). New screening methods for probiotics with adhesion properties to sialic acid and sulphate residues in human colonic mucin using the Biacore assay. J. Appl. Microbiol..

[21] Uchida H., Fujitani K., Kawai Y., Kitazawa H., Horii A., Shiiba K., Saito K., Saito T. (2004). A New Assay Using Surface Plasmon Resonance (SPR) to Determine Binding of the Lactobacillus acidophilus Group to Human Colonic Mucin. Biosci. Biotechnol. Biochem..

[22] Kinoshita H., Wakahara N., Watanabe M., Kawasaki T., Matsuo H., Kawai Y., Kitazawa H., Ohnuma S., Miura K., Horii A. (2008). Cell surface glyceraldehyde-3-phosphate dehydrogenase (GAPDH) of Lactobacillus plantarum LA 318 recognizes human A and B blood group antigens. Res. Microbiol..

[23] Zhou B., Albarracin L., Masumizu Y., Indo Y., Islam M.A., Garcia-Castillo V., Ikeda-Ohtsubo W., Suda Y., Aso H., Villena J. (2020). Draft Genome Sequence of Ligilactobacillus salivarius FFIG58, Isolated from the Intestinal Tract of Wakame-Fed Pig. Microbiol. Resour. Announc..

[24] Audisio M.C., Albarracín L., Torres M.J., Saavedra L., Hebert E.M., Villena J. (2018). Draft Genome Sequences of *Lactobacillus salivarius* A3iob and *Lactobacillus johnsonii* CRL1647, Novel Potential Probiotic Strains for Honeybees (*Apis mellifera* L.). Microbiol. Resour. Announc..

[25] Tatusova T., Dicuccio M., Badretdin A., Chetvernin V., Nawrocki E.P., Zaslavsky L., Lomsadze A., Pruitt K.D., Borodovsky M., Ostell J. (2016). NCBI prokaryotic genome annotation pipeline. Nucleic Acids Res..

[26] Aziz R.K., Bartels D., Best A.A., DeJongh M., Disz T., Edwards R.A., Formsma K., Gerdes S., Glass E.M., Kubal M. (2008). The RAST Server: Rapid Annotations using Subsystems Technology. BMC Genom..

[27] Rodriguez-R L., Konstantinidis K. (2016). The enveomics collection: A toolbox for specialized analyses of microbial genomes and metagenomes. PeerJ Prepr..

[28] Edgar R.C. (2004). MUSCLE: Multiple sequence alignment with high accuracy and high throughput. Nucleic Acids Res..

[29] Kumar S., Stecher G., Li M., Knyaz C., Tamura K. (2018). MEGA X: Molecular evolutionary genetics analysis across computing platforms. Mol. Biol. Evol..

[30] Saitou N., Nei M. (1987). The neighbor-joining method: A new method for reconstructing phylogenetic trees. Mol. Biol. Evol..

[31] Tamura K., Nei M., Kumar S. (2004). Prospects for inferring very large phylogenies by using the neighbor-joining method. Proc. Natl. Acad. Sci. USA.

[32] Warnes G.R., Bolker B., Bonebakker L., Gentleman R., Liaw W.H.A., Lumley T., Maechler M., Magnusson A., Moeller S., Schwartz M. (2015). Package “gplots”: Various R Programming Tools for Plotting Data.

[33] Page A.J., Cummins C.A., Hunt M., Wong V.K., Reuter S., Holden M.T.G., Fookes M., Falush D., Keane J.A., Parkhill J. (2015). Roary: Rapid large-scale prokaryote pan genome analysis. Bioinformatics.

[34] Seemann T. (2014). Prokka: Rapid prokaryotic genome annotation. Bioinformatics.

[35] Heberle H., Meirelles V.G., da Silva F.R., Telles G.P., Minghim R. (2015). InteractiVenn: A web-based tool for the analysis of sets through Venn diagrams. BMC Bioinform..

[36] Ishizuka T., Kanmani P., Kobayashi H., Miyazaki A., Soma J., Suda Y., Aso H., Nochi T., Iwabuchi N., Xiao J. (2016). Immunobiotic Bifidobacteria Strains Modulate Rotavirus Immune Response in Porcine Intestinal Epitheliocytes via Pattern Recognition Receptor Signaling. PLoS ONE.

[37] Shimazu T., Villena J., Tohno M., Fujie H., Hosoya S., Shimosato T., Aso H., Suda Y., Kawai Y., Saito T. (2012). Immunobiotic Lactobacillus jensenii elicits anti-inflammatory activity in porcine intestinal epithelial cells by modulating negative regulators of the Toll-like receptor signaling pathway. Infect. Immun..

[38] Albarracin L., García-Castillo V., Masumizu Y., Indo Y., Islam M.A., Suda Y., Garcia A., Aso H., Takahashi H., Kitazawa H. (2020). Efficient selection of new immunobiotic strains with antiviral effects in local and distal mucosal sites by using porcine intestinal epitheliocytes. Front. Immunol..

[39] Richter M., Rosselló-Móra R. (2009). Shifting the genomic gold standard for the prokaryotic species definition. Proc. Natl. Acad. Sci. USA.

[40] Harris H.M.B., Bourin M.J.B., Claesson M.J., O’Toole P.W. (2017). Phylogenomics and comparative genomics of *Lactobacillus salivarius*, a mammalian gut commensal. Microb. Genom..

[41] Bernard E., Rolain T., David B., André G., Dupres V., Dufrêne Y.F., Hallet B., Chapot-Chartier M.P., Hols P. (2012). Dual Role for the O-Acetyltransferase OatA in Peptidoglycan Modification and Control of Cell Septation in Lactobacillus plantarum. PLoS ONE.

[42] Brott A.S., Clarke A.J. (2019). Peptidoglycan O-acetylation as a virulence factor: Its effect on lysozyme in the innate immune system. Antibiotics.

[43] Kolling Y., Salva S., Villena J., Alvarez S. (2018). Are the immunomodulatory properties of Lactobacillus rhamnosus CRL1505 peptidoglycan common for all Lactobacilli during respiratory infection in malnourished mice?. PLoS ONE.

[44] Clua P., Tomokiyo M., Raya Tonetti F., Islam M.A., García Castillo V., Marcial G., Salva S., Alvarez S., Takahashi H., Kurata S. (2020). The Role of Alveolar Macrophages in the Improved Protection against Respiratory Syncytial Virus and Pneumococcal Superinfection Induced by the Peptidoglycan of Lactobacillus rhamnosus CRL1505. Cells.

[45] Mizuno H., Arce L., Tomotsune K., Albarracin L., Funabashi R., Vera D., Islam M.A., Vizoso-Pinto M.G., Takahashi H., Sasaki Y. (2020). Lipoteichoic Acid Is Involved in the Ability of the Immunobiotic Strain Lactobacillus plantarum CRL1506 to Modulate the Intestinal Antiviral Innate Immunity Triggered by TLR3 Activation. Front. Immunol..

[46] Raftis E.J., Forde B.M., Claesson M.J., O’Toole P.W. (2014). Unusual genome complexity in *Lactobacillus salivarius* JCM1046. BMC Genom..

[47] Liu C.T., Hsu I.T., Chou C.C., Lo P.R., Yu R.C. (2009). Exopolysaccharide production of *Lactobacillus salivarius* BCRC 14759 and bifidobacterium bifidum BCRC 14615. World J. Microbiol. Biotechnol..

[48] Kanmani P., Albarracin L., Kobayashi H., Iida H., Komatsu R., Humayun Kober A.K.M., Ikeda-Ohtsubo W., Suda Y., Aso H., Makino S. (2018). Exopolysaccharides from Lactobacillus delbrueckii OLL1073R-1 modulate innate antiviral immune response in porcine intestinal epithelial cells. Mol. Immunol..

[49] Granato D., Perotti F., Masserey I., Rouvet M., Golliard M., Servin A., Brassart D. (1999). Cell surface-associated lipoteichoic acid acts as an adhesion factor for attachment of Lactobacillus johnsonii La1 to human enterocyte-like Caco-2 cells. Appl. Environ. Microbiol..

[50] Živković M., Miljković M.S., Ruas-Madiedo P., Markelić M.B., Veljović K., Tolinački M., Soković S., Korać A., Golić N. (2016). EPS-SJ exopolisaccharide produced by the strain Lactobacillus paracasei subsp. paracasei BGSJ2-8 is involved in adhesion to epithelial intestinal cells and decrease on E. coli association to Caco-2 cells. Front. Microbiol..

[51] Kleerebezem M., Hols P., Bernard E., Rolain T., Zhou M., Siezen R.J., Bron P.A. (2010). The extracellular biology of the lactobacilli. FEMS Microbiol. Rev..

[52] Chen I., Dubnau D. (2004). DNA uptake during bacterial transformation. Nat. Rev. Microbiol..

[53] Cross B.W., Ruhl S. (2018). Glycan recognition at the saliva—Oral microbiome interface. Cell. Immunol..

[54] Chen Q., Sun B., Wu H., Peng Z., Fives-Taylor P.M. (2007). Differential roles of individual domains in selection of secretion route of a Streptococcus parasanguinis serine-rich adhesin, Fap1. J. Bacteriol..

[55] Zhou M., Wu H. (2009). Glycosylation and biogenesis of a family of serine-rich bacterial adhesins. Microbiology.

[56] Bensing B.A., Seepersaud R., Yen Y.T., Sullam P.M. (2014). Selective transport by SecA2: An expanding family of customized motor proteins. Biochim. Biophys. Acta Mol. Cell Res..

[57] Frese S.A., MacKenzie D.A., Peterson D.A., Schmaltz R., Fangman T., Zhou Y., Zhang C., Benson A.K., Cody L.A., Mulholland F. (2013). Molecular characterization of host-specific biofilm formation in a vertebrate gut symbiont. PLoS Genet..

[58] De Boeck I., van den Broek M.F.L., Allonsius C.N., Spacova I., Wittouck S., Martens K., Wuyts S., Cauwenberghs E., Jokicevic K., Vandenheuvel D. (2020). Lactobacilli have a niche in the human nose. Cell Rep..

[59] Couvigny B., Lapaque N., Rigottier-Gois L., Guillot A., Chat S., Meylheuc T., Kulakauskas S., Rohde M., Mistou M.Y., Renault P. (2017). Three glycosylated serine-rich repeat proteins play a pivotal role in adhesion and colonization of the pioneer commensal bacterium, *Streptococcus salivarius*. Environ. Microbiol..

[60] Couvigny B., Kulakauskas S., Pons N., Quinquis B., Abraham A.L., Meylheuc T., Delorme C., Renault P., Briandet R., Lapaque N. (2018). Identification of new factors modulating adhesion abilities of the pioneer commensal bacterium Streptococcus salivarius. Front. Microbiol..

[61] Feltcher M.E., Braunstein M. (2012). Emerging themes in SecA2-mediated protein export. Nat. Rev. Microbiol..

[62] Wegmann U., MacKenzie D.A., Zheng J., Goesmann A., Roos S., Swarbreck D., Walter J., Crossman L.C., Juge N. (2015). The pan-genome of Lactobacillus reuteri strains originating from the pig gastrointestinal tract. BMC Genom..

[63] Frese S.A., Benson A.K., Tannock G.W., Loach D.M., Kim J., Zhang M., Oh P.L., Heng N.C.K., Patil P.B., Juge N. (2011). The evolution of host specialization in the vertebrate gut symbiont *Lactobacillus reuteri*. PLoS Genet..

[64] Wuyts S., Wittouck S., De Boeck I., Allonsius C.N., Pasolli E., Segata N., Lebeer S. (2017). Large-Scale Phylogenomics of the Lactobacillus casei Group Highlights Taxonomic Inconsistencies and Reveals Novel Clade-Associated Features. mSystems.

[65] Latousakis D., Juge N. (2018). How sweet are our gut beneficial bacteria? A focus on protein glycosylation in Lactobacillus. Int. J. Mol. Sci..

[66] MacKenzie D.A., Tailford L.E., Hemmings A.M., Juge N. (2009). Crystal structure of a mucus-binding protein repeat reveals an unexpected functional immunoglobulin binding activity. J. Biol. Chem..

[67] Etzold S., Kober O.I., Mackenzie D.A., Tailford L.E., Gunning A.P., Walshaw J., Hemmings A.M., Juge N. (2014). Structural basis for adaptation of lactobacilli to gastrointestinal mucus. Environ. Microbiol..

[68] Etzold S., Juge N. (2014). Structural insights into bacterial recognition of intestinal mucins. Curr. Opin. Struct. Biol..

[69] Gunning A.P., Kavanaugh D., Thursby E., Etzold S., Mackenzie D.A., Juge N. (2016). Use of atomic force microscopy to study the multi-modular interaction of bacterial adhesins to mucins. Int. J. Mol. Sci..

[70] Kankainen M., Paulin L., Tynkkynen S., Von Ossowski I., Reunanen J., Partanen P., Satokari R., Vesterlund S., Hendrickx A.P.A., Lebeer S. (2009). Comparative genomic analysis of Lactobacillus rhamnosus GG reveals pili containing a human-mucus binding protein. Proc. Natl. Acad. Sci. USA.

[71] Von Ossowski I. (2017). Novel molecular insights about lactobacillar sortase-dependent piliation. Int. J. Mol. Sci..

[72] Edelman S.M., Lehti T.A., Kainulainen V., Antikainen J., Kylväjä R., Baumann M., Westerlund-Wikström B., Korhonen T.K. (2012). Identification of a high-molecular-mass Lactobacillus epithelium adhesin (LEA) of Lactobacillus crispatus ST1 that binds to stratified squamous epithelium. Microbiology (United Kingdom).

[73] Ojala T., Kuparinen V., Koskinen J.P., Alatalo E., Holm L., Auvinen P., Edelman S., Westerlund-Wikström B., Korhonen T.K., Paulin L. (2010). Genome sequence of Lactobacillus crispatus ST1. J. Bacteriol..

[74] Marcotte H., Krogh Andersen K., Lin Y., Zuo F., Zeng Z., Larsson P.G., Brandsborg E., Brønstad G., Hammarström L. (2017). Characterization and complete genome sequences of L. rhamnosus DSM 14870 and L. gasseri DSM 14869 contained in the EcoVag^®^ probiotic vaginal capsules. Microbiol. Res..

[75] Pantoja-Feliciano I.G., Clemente J.C., Costello E.K., Perez M.E., Blaser M.J., Knight R., Dominguez-Bello M.G. (2013). Biphasic assembly of the murine intestinal microbiota during early development. ISME J..

[76] Dominguez-Bello M.G., De Jesus-Laboy K.M., Shen N., Cox L.M., Amir A., Gonzalez A., Bokulich N.A., Song S.J., Hoashi M., Rivera-Vinas J.I. (2016). Partial restoration of the microbiota of cesarean-born infants via vaginal microbial transfer. Nat. Med..

[77] Kmet V., Lucchini F. (1999). Aggregation of sow lactobacilli with diarrhoeagenic *Escherichia coli*. J. Vet. Med. Ser. B.

[78] Martín R., Delgado S., Maldonado A., Jiménez E., Olivares M., Fernández L., Sobrino O.J., Rodríguez J.M. (2009). Isolation of lactobacilli from sow milk and evaluation of their probiotic potential. J. Dairy Res..

