# Supplementary material for: Selection of Immunobiotic Ligilactobacillus salivarius Strains from the Intestinal Tract of Wakame-Fed Pigs: Functional and Genomic Studies
Source: Microorganisms. 2020 Oct 26;8(11):1659. doi: 10.3390/microorganisms8111659 (PMC7716343; doi:10.3390/microorganisms8111659)
Supplement: Supplementary file 1 [file microorganisms-08-01659-s001.zip › Trab ZHOU FINAL/Supplementary Table 3.docx]

| ***Ligilactobacillus salivarius* strain** | **Host** | **Sample** | **Genome size (bp)** | **G+C content (%)** | **Protein-coding genes** | **GenBank ID** |
| --- | --- | --- | --- | --- | --- | --- |
| A3iob | *Apis mellifera* | Intestine | 2,054,490 | 32.6 | 1,983 | QFAS00000000.1 |
| LPM01 | *Homo sapiens* | Milk | 1,788,723 | 33.0 | 1,717 | LT604074.1 |
| CECT 5713 | *Homo sapiens* | Milk | 1,828,169 | 32.9 | 1,884 | CP017107.1 |
| HN26-4 | *Homo sapiens* | Intestine | 1,953,911 | 32.6 | 1,811 | VSTO00000000.1 |
| FXJCJ7_2 | *Homo sapiens* | Intestine | 1,891,266 | 32.8 | 1,730 | VSUK00000000.1 |
| NT4-8 | *Homo sapiens* | Intestine | 1,910,114 | 32.7 | 1,802 | VSTK00000000.1 |
| JCM1046 | *Sus scrofa* | Intestine | 1,836,297 | 33.1 | 1,803 | CP007646.1 |
| ZLS006 | *Sus scrofa* | Intestine | 2,177,581 | 33.2 | 2,114 | CP020858.1 |
| KLA006 | *Sus scrofa* | Intestine | 2,366,896 | 32.9 | 2,276 | LXZO00000000.1 |
| KLF003 | *Sus scrofa* | Intestine | 2,206,918 | 32.7 | 2,117 | LXZL00000000.1 |
| KLW010 | *Sus scrofa* | Intestine | 2,389,395 | 32.7 | 2,255 | LXYX00000000.1 |
| cp400 | *Sus scrofa* | Intestine | 2,156,840 | 32.9 | 1,958 | CBVR000000000.1 |
| CICC 23174 | *Gallus gallus* | Intestine | 1,746,897 | 33.0 | 1,606 | CP002034.1 |
| DJ-sa-01 | *Gallus gallus* | Intestine | 1,870,629 | 33.0 | 1,719 | CP029616.1 |
| UCC118 | *Homo sapiens* | Intestine | 1,827,111 | 32.9 | 1,807 | CP000233.1 |
| REN | *Homo sapiens* | Intestine | 1,928,516 | 32.9 | 1,861 | CP011403.1 |

**Table Supplementary 3.** Comparison of the general genome features of sequenced *Ligilactobacillus salivarius* strains with public available genomes. *L. salivarius g*enomes were obtained from NCBI database.
