# Supplementary material for: Selection of Immunobiotic Ligilactobacillus salivarius Strains from the Intestinal Tract of Wakame-Fed Pigs: Functional and Genomic Studies
Source: Microorganisms. 2020 Oct 26;8(11):1659. doi: 10.3390/microorganisms8111659 (PMC7716343; doi:10.3390/microorganisms8111659)
Supplement: Supplementary file 1 [file microorganisms-08-01659-s001.zip › Trab ZHOU FINAL/Table 1.docx]

| ***Ligilactobacillus salivarius* strain** | **Host** | **Sample** | **Genome size**  **(bp)** | **G+C content (%)** | **Protein-coding genes** | **GenBank ID** | **Reference** |
| --- | --- | --- | --- | --- | --- | --- | --- |
| FFIG58 | *Sus scrofa* | Intestine | 1,984,018 | 32.8 | 1,891 | JACBJR000000000.1 | [20] |
| FFIG23 | *Sus scrofa* | Intestine | 2,041,027 | 32.8 | 1,932 | JACBJS000000000.1 | This work |
| FFIG53 | *Sus scrofa* | Intestine | 1,948,231 | 32.9 | 1,863 | JACBJT000000000.1 | This work |
| FFIG60 | *Sus scrofa* | Intestine | 1,948,639 | 33.0 | 1,855 | JACBJU000000000.1 | This work |
| FFIG63 | *Sus scrofa* | Intestine | 1,777,024 | 33.3 | 1,786 | JACBJV000000000.1 | This work |
| FFIG79 | *Sus scrofa* | Intestine | 1,718,597 | 33.4 | 1,728 | JACBJW000000000.1 | This work |
| FFIG124 | *Sus scrofa* | Intestine | 1,806,583 | 33.2 | 1,812 | JACBJX000000000.1 | This work |
| FFIG130 | *Sus scrofa* | Intestine | 1,862,635 | 33.1 | 1,778 | JACBJY000000000.1 | This work |

**Table 1.** General genomic features of *Ligilactobacillus salivarius* strains isolated from the intestinal tract of wakame-fed pig.
