# Supplementary material for: Selection of Immunobiotic Ligilactobacillus salivarius Strains from the Intestinal Tract of Wakame-Fed Pigs: Functional and Genomic Studies
Source: Microorganisms. 2020 Oct 26;8(11):1659. doi: 10.3390/microorganisms8111659 (PMC7716343; doi:10.3390/microorganisms8111659)
Supplement: Supplementary file 1 [file microorganisms-08-01659-s001.zip › Trab ZHOU FINAL/Supplementary figures.docx]

**Selection of immunobiotic *Ligilactobacillus salivarius* strains from the intestinal tract of wakame-fed pig: functional and genomic studies**


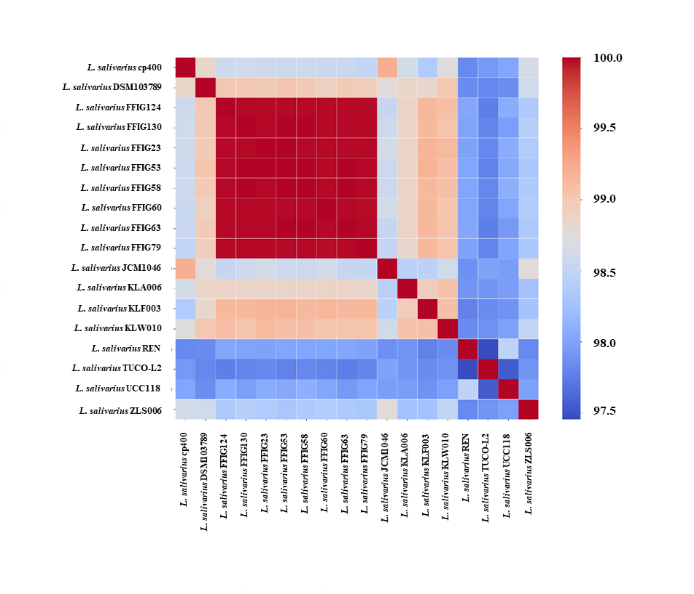


**Figure Supplementary 1.** Clustering of pair-wise average nucleotide identity (ANI) scores of *Ligilactobacillus salivarius* strains isolated from the intestinal mucosa of wakame-fed pigs and compared with *L. salivarius* strains with public available complete genomes.


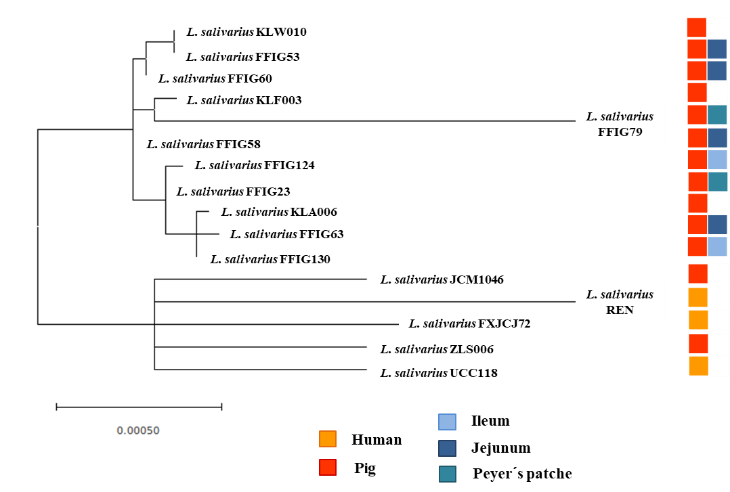


**Figure Supplementary 2.** Hierarchical clustering of *Ligilactobacillus salivarius* strains isolated from the intestinal mucosa of wakame-fed pigs. The phylogenetic trees were constructed based on the 16s RNA extracted from the genomes of FFIG strains as well as from *L. salivarius* strains with public available complete genomes.

**
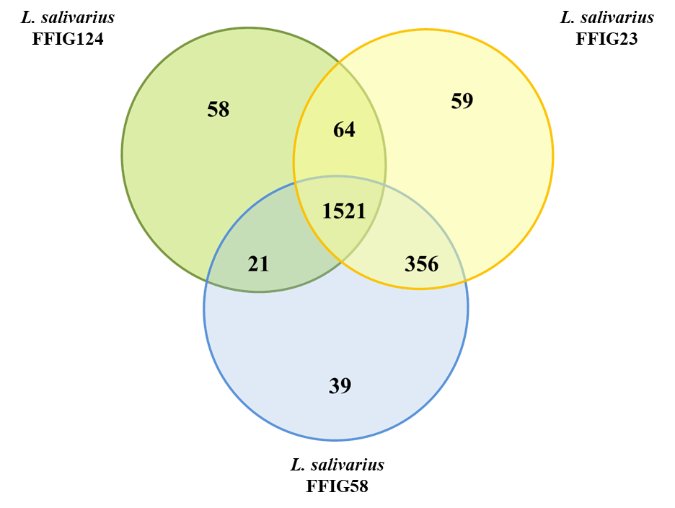
**

**Figure Supplementary 3.** Genomic comparison of *Ligilactobacillus salivarius* FFIG58, FFIG23 and FFIG124 isolated from the intestinal mucosa of wakame-fed pigs. Venn diagram depict the number of unique genes in each genome and the numbers of gene sheared by the strains.

**
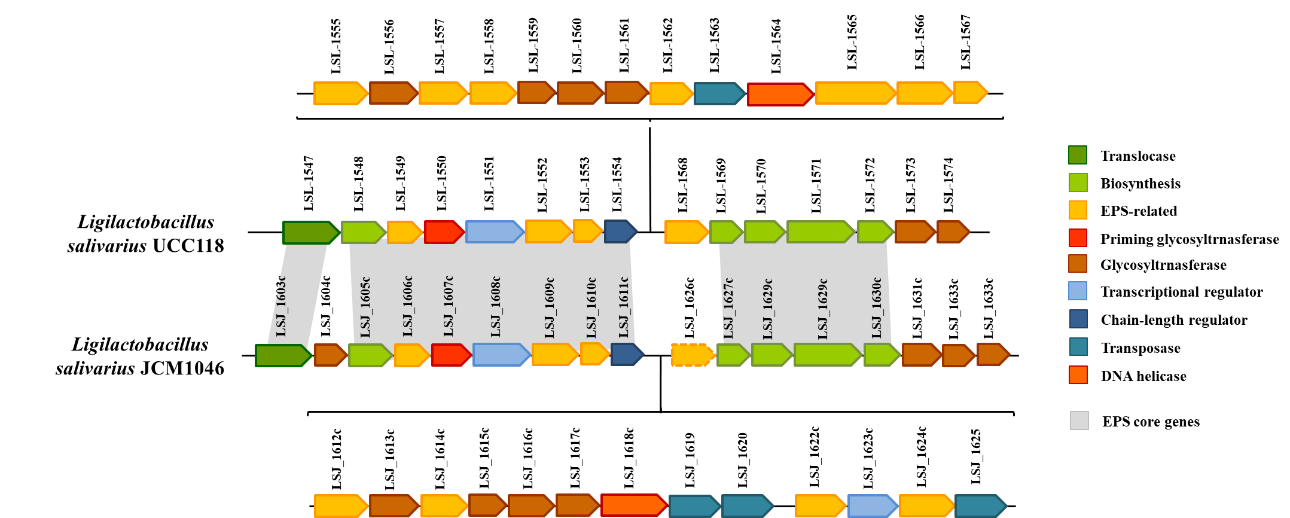
**

**Figure Supplementary 4.** Comparison of the exopolysaccharide (EPS) clusters from *Ligilactobacillus salivarius* UCC118 and JCM1046. The genes sheared by the two strains (grey) are conserved and were considered as EPS core genes. Dotted lines indicate non-functional genes.


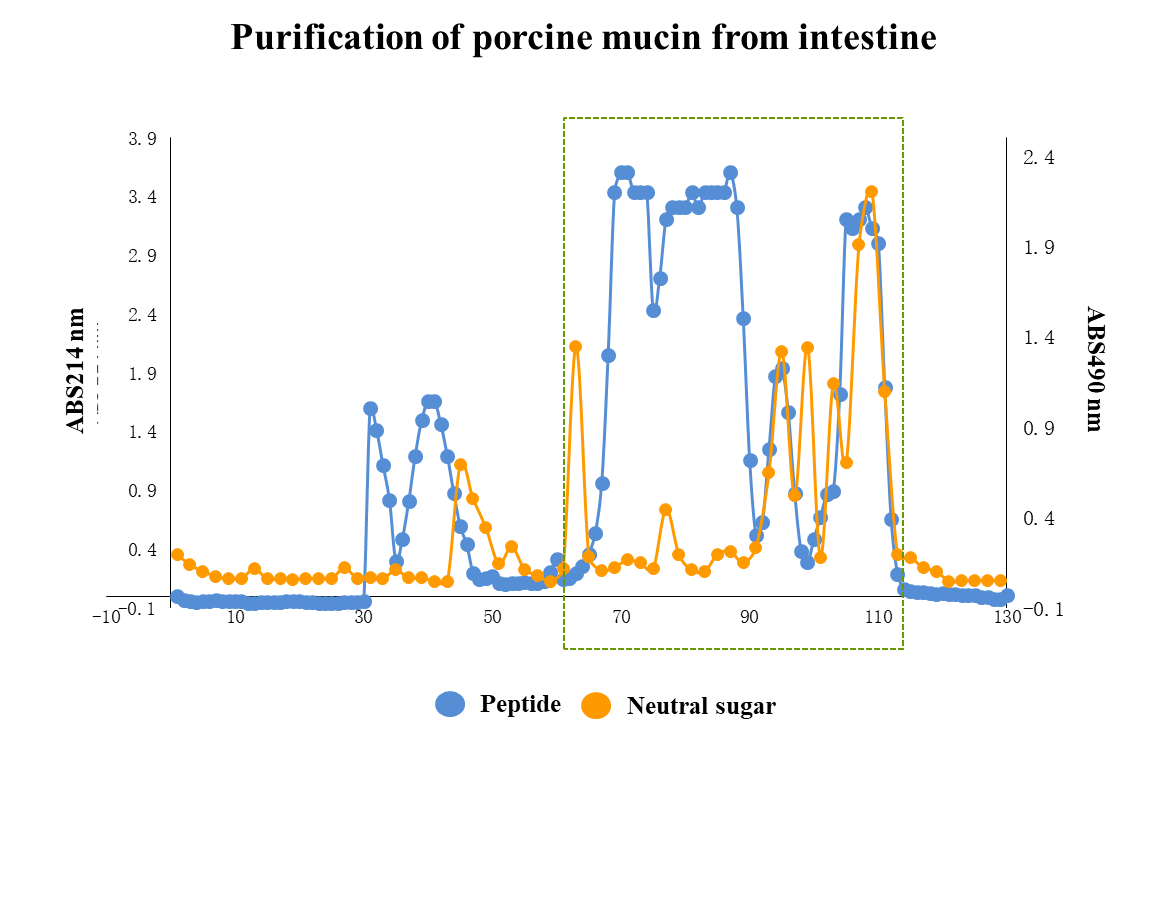


**Figure Supplementary 5.** Purification of porcine mucins for the evaluation of adhesion of *Ligilactobacillus salivarius* strains isolated from the intestinal mucosa of wakame-fed pigs.


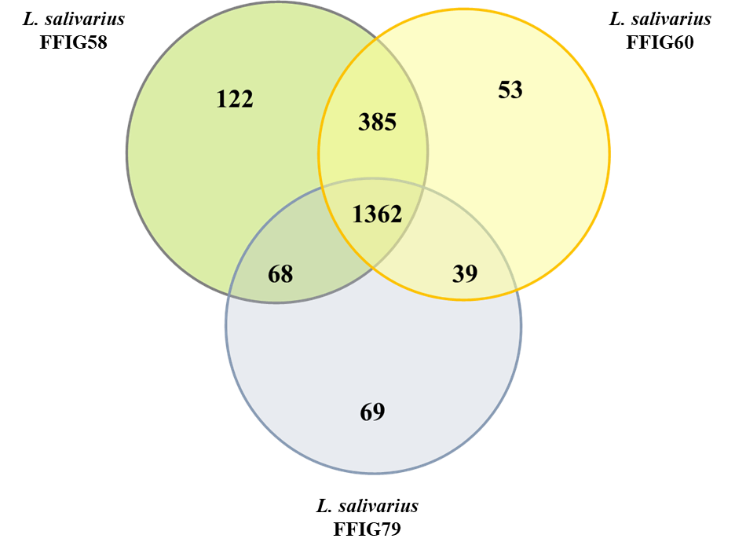


**Figure Supplementary 6.** Genomic comparison of *Ligilactobacillus salivarius* strains isolated from the intestinal mucosa of wakame-fed pigs. Three “adhesion phenotypes” defined according to the ability of *L. salivarius* FFIG strains to adhere to porcine and human mucins and to porcine intestinal epithelial (PIE) cells were compared. *L. salivarius* FFIG58, FFIG60 and FFIG79. Venn diagram depict the number of unique genes in each genome and the numbers of gene sheared by the strains.


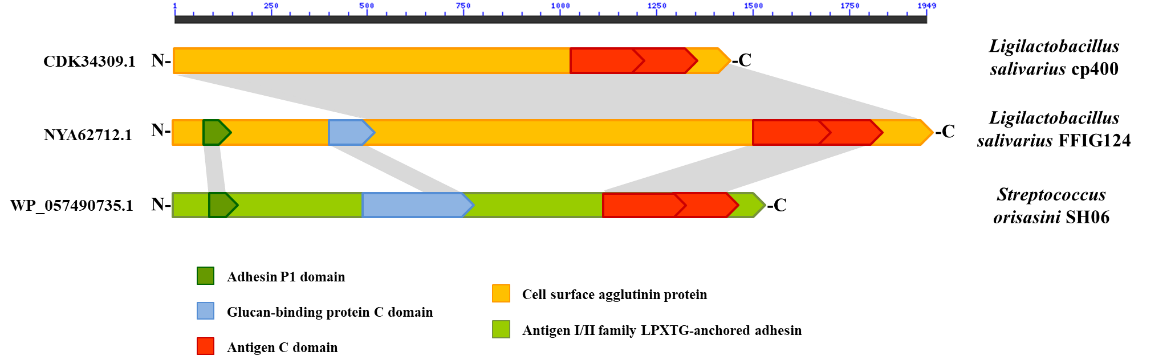


**Figure Supplementary 7.** Comparison of the domain organization in the putative cell surface agglutinin proteins from *Ligilactobacillus salivarius* FFIG124 and cp400 and the antigen I/II family LPXTG-anchored adhesin from *Streptococcus orisanini* SH06. The antigen I/II family LPXTG-anchored adhesin is a protein of 1,518 amino acids while the putative cell surface agglutinin proteins from the FFIG124 and cp400 strains are proteins of 1,949 and 1,420 amino acids, respectively. Conserved amino acids sequences are shown in gray.


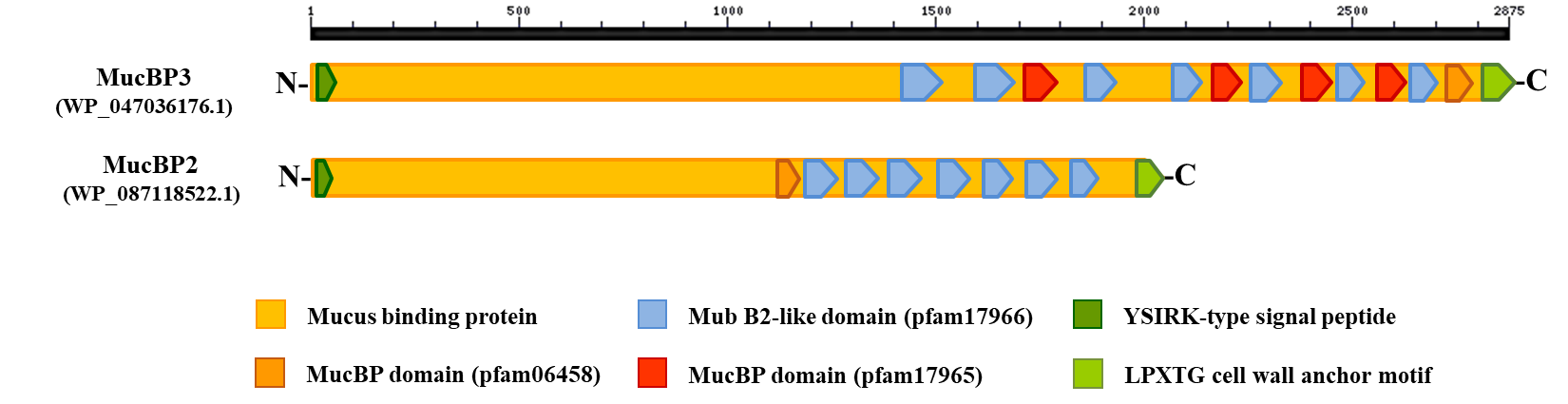


**Figure Supplementary 8.** Comparison of the domain organization of mucus binding proteins (MucBP) from *Ligilactobacillus salivarius* strains isolated from the intestinal mucosa of wakame-fed pigs.
