# Supplementary material for: Selection of Immunobiotic Ligilactobacillus salivarius Strains from the Intestinal Tract of Wakame-Fed Pigs: Functional and Genomic Studies
Source: Microorganisms. 2020 Oct 26;8(11):1659. doi: 10.3390/microorganisms8111659 (PMC7716343; doi:10.3390/microorganisms8111659)
Supplement: Supplementary file 1 [file microorganisms-08-01659-s001.zip › Trab ZHOU FINAL/Supplementary Table 1.docx]

**Table Supplementary 1.** *Ligilactobacillus salivarius* strains evaluated in this study.

| No. | Strains | Species | Origin (porcine intestine) |
| --- | --- | --- | --- |
| 1 | FFIG17 | *Ligilactobacillus salivarius* | ileum peyer's patch |
| 2 | FFIG18 | *Ligilactobacillus salivarius* | ileum peyer's patch |
| 3 | FFIG19 | *Ligilactobacillus salivarius* | ileum peyer's patch |
| 4 | FFIG20 | *Ligilactobacillus salivarius* | ileum peyer's patch |
| 5 | FFIG21 | *Ligilactobacillus salivarius* | ileum peyer's patch |
| 6 | FFIG22 | *Ligilactobacillus salivarius* | ileum peyer's patch |
| 7 | FFIG23 | *Ligilactobacillus salivarius* | ileum peyer's patch |
| 8 | FFIG24 | *Ligilactobacillus salivarius* | ileum peyer's patch |
| 9 | FFIG26 | *Ligilactobacillus salivarius* | ileum peyer's patch |
| 10 | FFIG27 | *Ligilactobacillus salivarius* | ileum peyer's patch |
| 11 | FFIG28 | *Ligilactobacillus salivarius* | ileum peyer's patch |
| 12 | FFIG29 | *Ligilactobacillus salivarius* | ileum peyer's patch |
| 13 | FFIG30 | *Ligilactobacillus salivarius* | ileum peyer's patch |
| 14 | FFIG31 | *Ligilactobacillus salivarius* | ileum peyer's patch |
| 15 | FFIG32 | *Ligilactobacillus salivarius* | ileum peyer's patch |
| 16 | FFIG33 | *Ligilactobacillus salivarius* | ileum peyer's patch |
| 17 | FFIG34 | *Ligilactobacillus salivarius* | ileum peyer's patch |
| 18 | FFIG35 | *Ligilactobacillus salivarius* | ileum peyer's patch |
| 19 | FFIG36 | *Ligilactobacillus salivarius* | ileum peyer's patch |
| 20 | FFIG37 | *Ligilactobacillus salivarius* | ileum peyer's patch |
| 21 | FFIG38 | *Ligilactobacillus salivarius* | ileum peyer's patch |
| 22 | FFIG39 | *Ligilactobacillus salivarius* | ileum peyer's patch |
| 23 | FFIG40 | *Ligilactobacillus salivarius* | ileum peyer's patch |
| 24 | FFIG41 | *Ligilactobacillus salivarius* | ileum peyer's patch |
| 25 | FFIG42 | *Ligilactobacillus salivarius* | ileum peyer's patch |
| 26 | FFIG43 | *Ligilactobacillus salivarius* | ileum peyer's patch |
| 27 | FFIG44 | *Ligilactobacillus salivarius* | ileum peyer's patch |
| 28 | FFIG45 | *Ligilactobacillus salivarius* | ileum peyer's patch |
| 29 | FFIG46 | *Ligilactobacillus salivarius* | ileum peyer's patch |
| 30 | FFIG47 | *Ligilactobacillus salivarius* | jejunum |
| 31 | FFIG48 | *Ligilactobacillus salivarius* | jejunum |
| 32 | FFIG49 | *Ligilactobacillus salivarius* | jejunum |
| 33 | FFIG50 | *Ligilactobacillus salivarius* | jejunum |
| 34 | FFIG51 | *Ligilactobacillus salivarius* | jejunum |
| 35 | FFIG52 | *Ligilactobacillus salivarius* | jejunum |
| 36 | FFIG53 | *Ligilactobacillus salivarius* | jejunum |
| 37 | FFIG54 | *Ligilactobacillus salivarius* | jejunum |
| 38 | FFIG55 | *Ligilactobacillus salivarius* | jejunum |
| 39 | FFIG56 | *Ligilactobacillus salivarius* | jejunum |
| 40 | FFIG57 | *Ligilactobacillus salivarius* | jejunum |
| 41 | FFIG58 | *Ligilactobacillus salivarius* | jejunum |
| 42 | FFIG59 | *Ligilactobacillus salivarius* | jejunum |
| 43 | FFIG60 | *Ligilactobacillus salivarius* | jejunum |
| 44 | FFIG61 | *Ligilactobacillus salivarius* | jejunum |
| 45 | FFIG62 | *Ligilactobacillus salivarius* | jejunum |
| 46 | FFIG63 | *Ligilactobacillus salivarius* | jejunum |
| 47 | FFIG64 | *Ligilactobacillus salivarius* | jejunum |
| 48 | FFIG65 | *Ligilactobacillus salivarius* | jejunum |
| 49 | FFIG66 | *Ligilactobacillus salivarius* | jejunum |
| 50 | FFIG67 | *Ligilactobacillus salivarius* | jejunum |
| 51 | FFIG68 | *Ligilactobacillus salivarius* | jejunum |
| 52 | FFIG69 | *Ligilactobacillus salivarius* | jejunum |
| 53 | FFIG70 | *Ligilactobacillus salivarius* | jejunum |
| 54 | FFIG71 | *Ligilactobacillus salivarius* | jejunum |
| 55 | FFIG72 | *Ligilactobacillus salivarius* | jejunum |
| 56 | FFIG73 | *Ligilactobacillus salivarius* | jejunum |
| 57 | FFIG74 | *Ligilactobacillus salivarius* | jejunum |
| 58 | FFIG75 | *Ligilactobacillus salivarius* | ileum peyer's patch |
| 59 | FFIG76 | *Ligilactobacillus salivarius* | ileum peyer's patch |
| 60 | FFIG77 | *Ligilactobacillus salivarius* | ileum peyer's patch |
| 61 | FFIG78 | *Ligilactobacillus salivarius* | ileum peyer's patch |
| 62 | FFIG79 | *Ligilactobacillus salivarius* | ileum peyer's patch |
| 63 | FFIG80 | *Ligilactobacillus salivarius* | ileum peyer's patch |
| 64 | FFIG81 | *Ligilactobacillus salivarius* | ileum peyer's patch |
| 65 | FFIG82 | *Ligilactobacillus salivarius* | ileum |
| 66 | FFIG83 | *Ligilactobacillus salivarius* | ileum |
| 67 | FFIG84 | *Ligilactobacillus salivarius* | ileum |
| 68 | FFIG85 | *Ligilactobacillus salivarius* | ileum |
| 69 | FFIG86 | *Ligilactobacillus salivarius* | ileum |
| 70 | FFIG87 | *Ligilactobacillus salivarius* | ileum |
| 71 | FFIG88 | *Ligilactobacillus salivarius* | ileum |
| 72 | FFIG89 | *Ligilactobacillus salivarius* | ileum |
| 73 | FFIG90 | *Ligilactobacillus salivarius* | ileum |
| 74 | FFIG91 | *Ligilactobacillus salivarius* | ileum |
| 75 | FFIG92 | *Ligilactobacillus salivarius* | ileum |
| 76 | FFIG93 | *Ligilactobacillus salivarius* | ileum |
| 77 | FFIG94 | *Ligilactobacillus salivarius* | ileum |
| 78 | FFIG95 | *Ligilactobacillus salivarius* | ileum |
| 79 | FFIG96 | *Ligilactobacillus salivarius* | ileum |
| 80 | FFIG97 | *Ligilactobacillus salivarius* | ileum |
| 81 | FFIG98 | *Ligilactobacillus salivarius* | ileum |
| 82 | FFIG99 | *Ligilactobacillus salivarius* | ileum |
| 83 | FFIG100 | *Ligilactobacillus salivarius* | ileum |
| 84 | FFIG101 | *Ligilactobacillus salivarius* | ileum |
| 85 | FFIG102 | *Ligilactobacillus salivarius* | ileum |
| 86 | FFIG103 | *Ligilactobacillus salivarius* | ileum |
| 87 | FFIG104 | *Ligilactobacillus salivarius* | ileum |
| 88 | FFIG105 | *Ligilactobacillus salivarius* | ileum |
| 89 | FFIG106 | *Ligilactobacillus salivarius* | ileum |
| 90 | FFIG107 | *Ligilactobacillus salivarius* | ileum |
| 91 | FFIG108 | *Ligilactobacillus salivarius* | ileum |
| 92 | FFIG109 | *Ligilactobacillus salivarius* | ileum |
| 94 | FFIG111 | *Ligilactobacillus salivarius* | ileum |
| 95 | FFIG112 | *Ligilactobacillus salivarius* | ileum |
| 96 | FFIG114 | *Ligilactobacillus salivarius* | ileum |
| 97 | FFIG116 | *Ligilactobacillus salivarius* | ileum |
| 98 | FFIG117 | *Ligilactobacillus salivarius* | ileum |
| 99 | FFIG118 | *Ligilactobacillus salivarius* | ileum |
| 100 | FFIG119 | *Ligilactobacillus salivarius* | ileum |
| 101 | FFIG120 | *Ligilactobacillus salivarius* | ileum |
| 102 | FFIG121 | *Ligilactobacillus salivarius* | ileum |
| 103 | FFIG122 | *Ligilactobacillus salivarius* | ileum |
| 104 | FFIG123 | *Ligilactobacillus salivarius* | ileum |
| 105 | FFIG125 | *Ligilactobacillus salivarius* | ileum |
| 106 | FFIG126 | *Ligilactobacillus salivarius* | ileum |
| 107 | FFIG127 | *Ligilactobacillus salivarius* | ileum |
| 108 | FFIG128 | *Ligilactobacillus salivarius* | ileum |
| 109 | FFIG129 | *Ligilactobacillus salivarius* | ileum |
| 110 | FFIG130 | *Ligilactobacillus salivarius* | ileum |
| 111 | FFIG131 | *Ligilactobacillus salivarius* | ileum |
| 112 | FFIG132 | *Ligilactobacillus salivarius* | ileum |
| 113 | FFIG133 | *Ligilactobacillus salivarius* | ileum |
| 114 | FFIG134 | *Ligilactobacillus salivarius* | ileum |
| 115 | FFIG135 | *Ligilactobacillus salivarius* | ileum |
| 116 | FFIG136 | *Ligilactobacillus salivarius* | ileum |
