# Supplementary material for: Selection of Immunobiotic Ligilactobacillus salivarius Strains from the Intestinal Tract of Wakame-Fed Pigs: Functional and Genomic Studies
Source: Microorganisms. 2020 Oct 26;8(11):1659. doi: 10.3390/microorganisms8111659 (PMC7716343; doi:10.3390/microorganisms8111659)
Supplement: Supplementary file 1 [file microorganisms-08-01659-s001.zip › Trab ZHOU FINAL/Supplementary Table 2.docx]

| ***Ligilactobacillus salivarius* strain** | **BioProject** | **BioSample** | **Contigs** | **tRNA-coding sequences** | **rRNA-coding sequences** | **Reference** |
| --- | --- | --- | --- | --- | --- | --- |
| FFIG58 | PRJNA643532 | SAMN15418202 | 222 | 36 | 11 | Zhou et al., 2020 |
| FFIG23 | PRJNA643821 | SAMN15430185 | 207 | 54 | 13 | This work |
| FFIG53 | PRJNA643821 | SAMN15430186 | 286 | 43 | 5 | This work |
| FFIG60 | PRJNA643821 | SAMN15430187 | 372 | 43 | 4 | This work |
| FFIG63 | PRJNA643821 | SAMN15430188 | 523 | 25 | 5 | This work |
| FFIG79 | PRJNA643821 | SAMN15430189 | 550 | 17 | 2 | This work |
| FFIG124 | PRJNA643821 | SAMN15430190 | 489 | 21 | 3 | This work |
| FFIG130 | PRJNA643821 | SAMN15430191 | 406 | 24 | 3 | This work |

**Table Supplementary 2.** General genomic features of *Ligilactobacillus salivarius* strains isolated from the intestinal tract of wakame-fed pig.
